# Dietary pterostilbene is a novel MTA1-targeted chemopreventive and therapeutic agent in prostate cancer

**DOI:** 10.18632/oncotarget.7841

**Published:** 2016-03-01

**Authors:** Swati Dhar, Avinash Kumar, Liangfen Zhang, Agnes M. Rimando, Janice M. Lage, Jack R. Lewin, Azeddine Atfi, Xu Zhang, Anait S. Levenson

**Affiliations:** ^1^ Cancer Institute, University of Mississippi Medical Center, Jackson, MS, USA; ^2^ Department of Pathology, University of Mississippi Medical Center, Jackson, MS, USA; ^3^ United States Department of Agriculture, Agriculture Research Service, Natural Product Utilization Research Unit, University, MS, USA; ^4^ Department of Biochemistry, University of Mississippi Medical Center, Jackson, MS, USA; ^5^ Center of Biostatistics and Bioinformatics, University of Mississippi Medical Center, Jackson, MS, USA; ^6^ Current affiliation: Arnold and Marie Schwartz College of Pharmacy and Health Sciences, Long Island University, Brooklyn, NY, USA

**Keywords:** pterostilbene, prostate cancer, chemoprevention, therapy, MTA1

## Abstract

Overexpression of the epigenetic modifier metastasis-associated protein 1 (MTA1) is associated with aggressive human prostate cancer. The purpose of this study was to determine MTA1- targeted chemopreventive and therapeutic efficacy of pterostilbene, a natural potent analog of resveratrol, in pre-clinical models of prostate cancer. Here, we show that high levels of MTA1 expression in *Pten*-loss prostate cooperate with key oncogenes, including c-Myc and Akt among others, to promote prostate cancer progression. Loss-of-function studies using human prostate cancer cells indicated direct involvement of MTA1 in inducing inflammation and epithelial-to-mesenchymal transition. Importantly, pharmacological inhibition of MTA1 by pterostilbene resulted in decreased proliferation and angiogenesis and increased apoptosis. This restrained prostatic intraepithelial neoplasia (PIN) formation in prostate-specific *Pten* heterozygous mice and reduced tumor development and progression in prostate-specific *Pten*-null mice. Our findings highlight MTA1 as a key upstream regulator of prostate tumorigenesis and cancer progression. More significantly, it offers pre-clinical proof for pterostilbene as a promising lead natural agent for MTA1-targeted chemopreventive and therapeutic strategy to curb prostate cancer.

## INTRODUCTION

Besides the role of genetic alterations in cancer, substantial data support the idea that dietary/lifestyle factors affect cancer initiation, promotion and progression through epigenetic alterations [1, 2]. Along these lines, anti-inflammatory and anti-oxidant epigenetic dietary agents are of great interest for cancer prevention and treatment, particularly for slow growing and age-related prostate cancer, for which diet is a risk factor [3].

One of the known epigenetic alterations in various cancers is overexpression of the master chromatin remodeler, metastasis-associated protein 1 (MTA1) [4], which correlates with higher grade tumor, recurrence, metastasis and poor prognosis [5–11]. MTA1 acts as a part of the NuRD co-repressor complex that is involved in histone deacetylation and gene silencing [4]. Alterations in MTA1 expression or regulation leads to deregulated chromatin, changes in gene transcription and/or inappropriate gene silencing [12].

Increased nuclear expression of MTA1 is associated with high Gleason score, aggressive disease, recurrence, and bone metastasis in human prostate cancer [8–10]. Our studies with prostate cancer cell lines and xenografts have highlighted the functional relevance of MTA1 in promoting tumor growth, invasion, angiogenesis and metastasis [9, 13]. While the exact molecular mechanisms that govern MTA1 activity in cancer are poorly understood, our group and others have showed that MTA1 can elicit anti-apoptotic effects through p53 deacetylation [14, 15] or pro-angiogenic actions via positive regulation of VEGF expression [9, 16]. In MTA1-expressing xenografts, we detected significantly higher tumor growth in PTEN-deficient LNCaP mice - compared to the PTEN-expressing DU145-xenografts [9]. This observation coupled with our recent findings of PTEN's deacetylation by MTA1 [17] prompted speculation that MTA1 might cooperate with PTEN loss for the establishment and progression of prostate cancer.

Although the link between diet, cancer and epigenetics is complex, epigenetic dietary agents are of great interest for cancer prevention and treatment [18]. Previously, we have shown that dietary stilbenes, commonly found in grapes and blueberries, inhibit MTA1 expression and function in prostate cancer [9, 13, 15]. Pterostilbene (PTER), a potent natural *trans*-3,5-dimethylether analog of resveratrol, based on its superior pharmacokinetic and pharmacodynamic properties [13, 19–22] appears the strongest candidate for clinical development.

In the current study, we evaluated the MTA1-targeted efficacy of pterostilbene in autochthonous prostate cancer pre-clinical models that harbor prostate-specific *Pten* heterozygous (*Pb-Cre4*; *Pten*^+/f^, hereafter referred as *Pten*^+/f^) and *Pten* knockout (*Pb-Cre4*; *Pten*^f/f^, hereafter referred as *Pten*^f/f^), which represent chemoprevention and intervention scenarios, respectively. We found that *Pten* loss resulted in a marked increase in MTA1 expression leading to activation of MTA1-dependent oncogenic and tumor progression-related signaling pathways. Importantly, pterostilbene both as dietary supplementation and interventional daily injections, through targeting MTA1 and its tumor promoting network, inhibited inflammation, proliferation and angiogenesis and induced apoptosis. These actions of pterostilbene resulted in reduction of prostatic intraepithelial neoplasia (PIN) lesions and adenocarcinomas in precancerous *Pten*^+/f^ and cancer-prone *Pten*^f/f^ models, respectively. We believe that our present findings of chemopreventive and therapeutic efficacy of an epigenetic dietary agent pterostilbene as a novel MTA1-targeted strategy, may have potential clinical applications in prostate cancer management.

## RESULTS

### *Pten* loss-induced MTA1 upregulation promotes prostate tumorigenesis and progression

We chose prostate-specific *Pten*-loss mouse models, represented by *Pten* heterozygous (*Pten*^+/f^) and *Pten*-null (*Pten*^f/f^) mice that are most suitable for testing novel chemopreventive and therapeutic options due to close resemblance to the human disease and an intact immune system. Based on our previous studies suggesting a possible inverse relationship between MTA1 and PTEN [9, 17], we hypothesized that MTA1-targeted therapy would be effective for blocking *Pten* loss-driven prostate tumor growth and progression.

**Figure 1:**
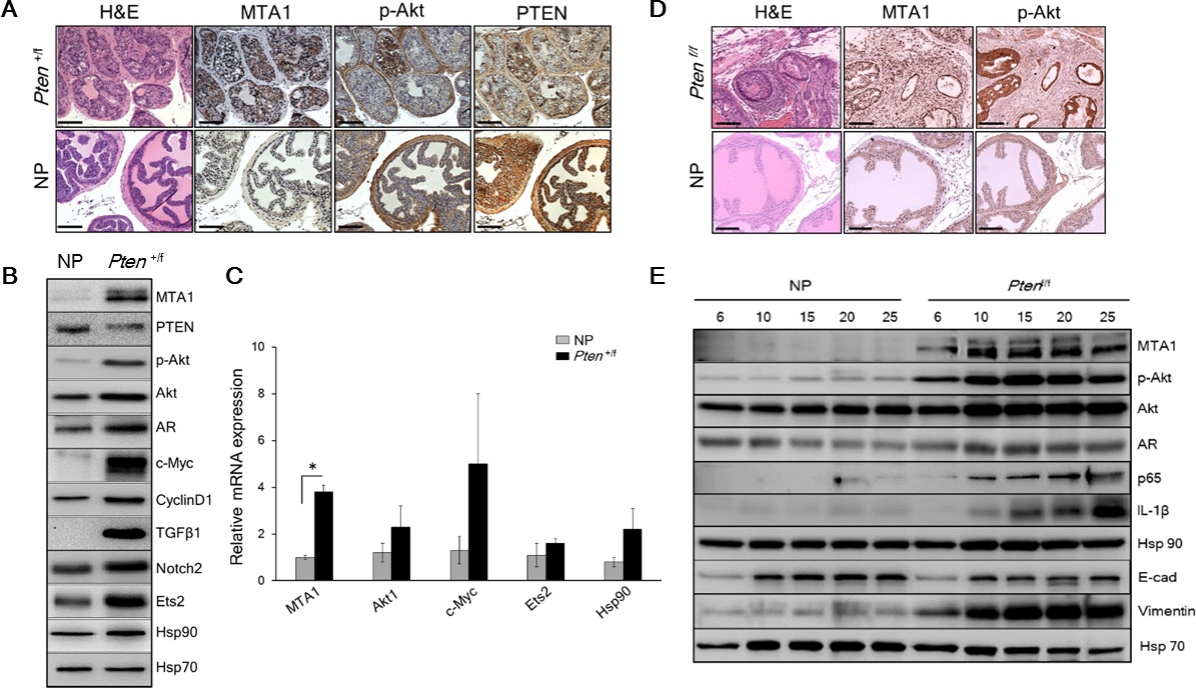
MTA1 promotes the *Pten* loss-driven prostate tumorigenesis and cancer progression (**A**) Comparison of H & E and IHC of MTA1, p-Akt and PTEN in the prostates from 10-month-old *Pten*^+/f^ mice and Cre-negative normal prostate (NP) controls. Scale bars, 100 μm. (**B**) Immunoblots of MTA1, PTEN, p-Akt, Akt, AR, c-Myc, CyclinD1, TGFβ1, Notch2, Ets2, and Hsp90 and (**C**) qRT-PCR analysis of MTA1, Akt1, c-Myc, Ets2 and Hsp90 mRNA levels in the prostate tissues from 10-month-old *Pten*^+/f^ mice compared to NP controls. (**D**) Comparison of H & E, MTA1, and p-Akt staining in the prostate tissues from 10-week-old *Pten*^f/f^ mice and NP controls. Scale bars, 100 μm. (**E**) Immunoblots of MTA1, p-Akt, Akt, AR, NF-κB (p65), IL-1β, Hsp90, E-cadherin (E-cad) and Vimentin in the prostate tissues from *Pten*^f/f^ mice compared to NP controls, isolated at the ages mentioned. Hsp70 was used as a loading control. qRT-PCR data represent the mean ± SEM (*n* = 3), *p < 0.05 (two-tailed, two-sample *t*-test).

To assess the possible involvement of MTA1 in *Pten* loss-driven prostate tumorigenesis, we examined MTA1 levels in prostate tissues from *Pten*^+/f^, which exhibit precancerous PIN at 8-10 months of age [23]. We found that loss of only one *Pten* allele was sufficient to substantially increase MTA1 both at protein and mRNA levels (Figure 1A–1C), suggesting that MTA1 may be involved in the initiation stage of prostate cancer.

Given that MTA1 has transcriptional co-repressor and co-activator functions [11, 24, 25], we hypothesized that MTA1 upregulation could transcriptionally perturb various genes and assist tumorigenesis promoting pathways. Our MTA1 ChIP-Seq analysis using prostates from 10-month-old *Pten*^+/f^ mice identified 38,000 peaks, including key oncogenes such as c-Myc, Akt1, Ets2, Notch2, CyclinD1 and Hsp90 [26–31] (Supplementary Table S1). Immunoblot analysis showed increased protein expression for these molecules as well as an increase in AR and TGFβ1 levels in the *Pten*^+/f^ prostates compared to normal prostates (Figure 1B). Quantitative real-time PCR (qRT-PCR) showing upregulation of Akt1, c-Myc, Ets2 and Hsp90 mRNA validated these genes as transcriptional targets of MTA1 (Figure 1C).

In the *Pten*^f/f^ (*Pten*-null) mice, which mimic stage-defined progression of human prostate cancer [23], we found an age-dependent increase in MTA1 expression along with expected increased levels of p-Akt as compared to their normal counterparts (Figure 1D and 1E), suggesting a strong correlation with the progression of prostate cancer. This notion has been supported by our previous studies of human specimens, in which MTA1 overexpression was correlated with prostate cancer progression, aggressiveness and metastasis [9, 10]. Immunohistochemical (IHC) analysis of the *Pten*-null prostates showed high MTA1 expression not only in the multilayers of luminal epithelial cells but also in the reactive stroma (Figure 1D). Since several factors and cytokines, including NF-κB (p65), IL-1β, and Hsp90 have been implicated either in MTA1 regulatory network or induction of reactive stroma or both [32–34], we found that concomitant with MTA1, the expression of these proteins was increased with continued tumor development (Figure 1E), suggesting a pro-inflammatory role for MTA1 in both the tumor and reactive stroma. Moreover, the expression of E-cadherin (an epithelial marker) was downregulated along with upregulation of Vimentin (a mesenchymal marker) (Figure 1E), indicating the involvement of MTA1 in epithelial-to-mesenchymal transition (EMT) of prostate cancer cells. Importantly, NF- κB (p65), IL-1β, Hsp90, E-cadherin and Vimentin were among the target gene promoters identified in our MTA1 ChIP-Seq analysis (Supplementary Table S1).

**Figure 2:**
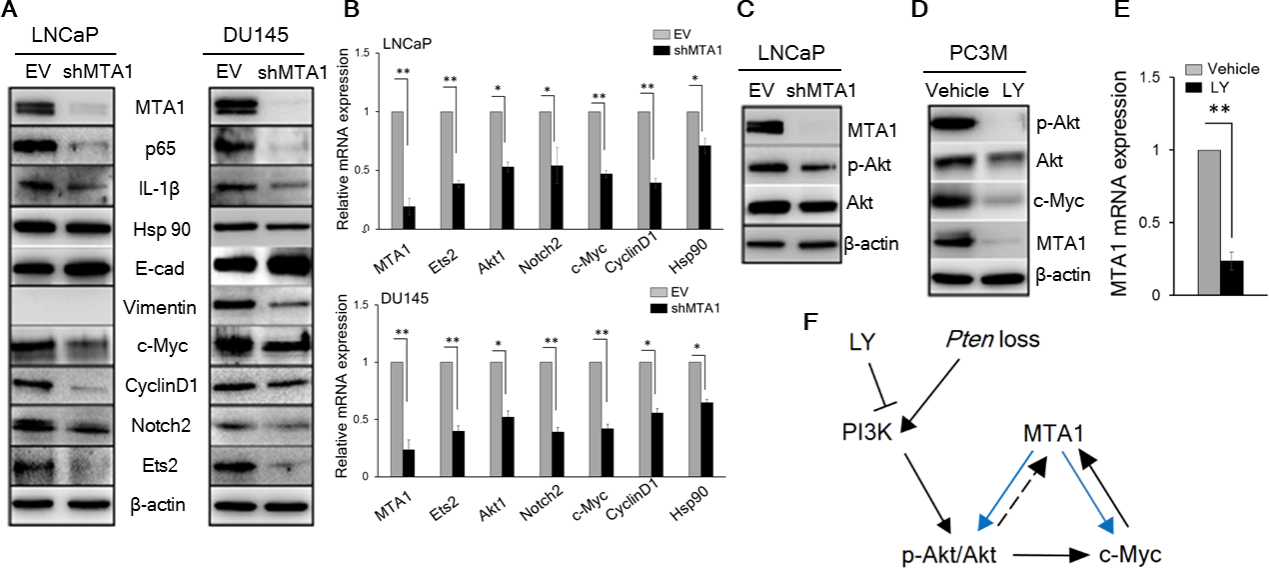
MTA1 directly regulates key molecular drivers of tumor promotion (**A**) Immunoblots of MTA1, NF-κB (p65), IL-1β, Hsp90, E-cadherin (E-cad), Vimentin, c-Myc, Cyclin D1, Notch2, and Ets2 in LNCaP (left) and DU145 (right) cells expressing (EV) and silenced for MTA1 (shMTA1). (**B**) qRT-PCR of MTA1, Ets2, Akt1, Notch2, c-Myc, Cyclin D1 and Hsp90 mRNA levels in LNCaP (top) and DU145 (bottom) EV and shMTA1 cells. (**C**) Immunoblot of MTA1, p-Akt and Akt in LNCaP EV and shMTA1 cells. (**D**) Immunoblot of p-Akt, Akt, c-Myc and MTA1 and (**E**) qRT-PCR of MTA1 mRNA levels in PC3M cells treated with vehicle (DMSO) and LY (LY294002). (**F**) Proposed mechanism involved in *Pten* loss-induced upregulation of MTA1, exhibiting the MTA1-Akt and MTA1-c-Myc feed-forward signaling loops (blue arrows), putative Akt-MTA1 link (dotted arrow). β-actin was used as a loading control. qRT-PCR data represent the mean ± SEM (*n* = 3), *p < 0.05; **p < 0.01 (two-tailed, two-sample *t*-test).

To gain insights into the mechanistic basis for the MTA1 upregulation in *Pten* loss-driven prostate tumorigenesis and tumor progression, we studied the effects of MTA1 knockdown in the human prostate cancer cell lines, LNCaP and DU145. We found a reduction in NF-κB (p65), IL-1β, and Vimentin and upregulation of E-cadherin protein levels in MTA1 knockdown (shMTA1) prostate cancer cells (Figure 2A and 2B), suggesting direct involvement of MTA1 in inflammation and EMT in prostate cancer. Likewise, we detected a reduction in the levels of c-Myc, CyclinD1, Notch2, Ets2, and Hsp90 both at protein and mRNA levels in shMTA1 cells (Figure 2A and 2B), suggesting a role for MTA1 upstream to the critical oncogenes c-Myc, Notch2 and Ets2. As expected, we found an activation of the Akt survival signaling pathway [23], which was accompanied by changes in AR levels in *Pten*^+/f^ and *Pten*^f/f^ mice (Figure 1A, 1B and 1D, 1E). The upregulation of Akt at both mRNA and protein levels was concomitant with increased MTA1 in the murine prostates (Figure 1B, 1C and 1E). Since Akt1 was among the MTA1 target promoters in the MTA1 ChIP-Seq analysis (Supplementary Table S1), we hypothesized possible direct association between MTA1 and Akt. Indeed, MTA1 depletion in *Pten*-deficient LNCaP cells led to partial inactivation of Akt through downregulation of Akt mRNA and protein (Figure 2B, top and 2C). Besides, inhibition of the Akt pathway by phosphatidylinositol 3-kinase (PI3K) inhibitor LY294002 led to reduced MTA1 protein and mRNA in PC3M cells (Figure 2D and 2E). Akt pathway is known to promote the stability of c-Myc protein [35], an identified transcriptional activator of MTA1 [36, 37]. We found that inhibition of Akt pathway led to a reduction in c-Myc protein levels (Figure 2D) but not mRNA (data not shown). On the other hand, the downregulation of MTA1 by LY294002 was both at protein and mRNA levels (Figure 2D and 2E), suggesting that the Akt may positively regulate MTA1 at the transcriptional level by promoting the stability of c-Myc protein (Figure 2F). Together, these results suggest a direct positive crosstalk between MTA1 and Akt pathway independent of PTEN.

**Figure 3:**
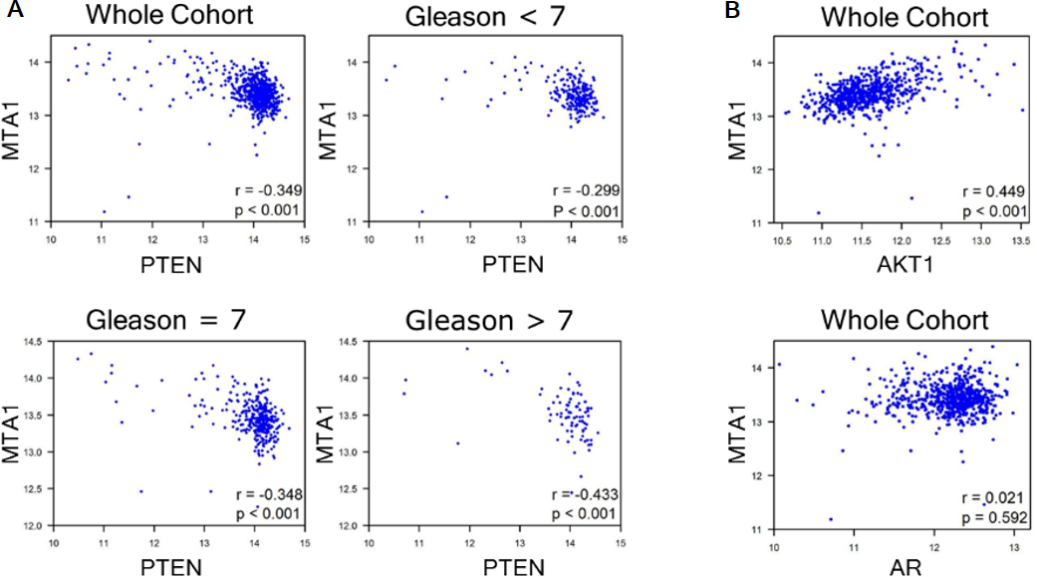
GEO analyses for correlation of MTA1 with PTEN, AKT1 and AR GSE41967 study of human prostate tissues (*n* = 639) [38] was used. Scatter plot depicting (**A**) strong negative correlation between MTA1 and PTEN (*r* = −0.349, whole cohort), which becomes stronger with increased Gleason score (*r* = −0.299, Gleason < 7; *r* = −0.348, Gleason = 7; *r* = −0.433, Gleason > 7, *p* < 0.001); (**B**) positive MTA1 correlation with AKT1 expression (*r* = 0.499, whole cohort, *p* < 0.001); and no correlation between MTA1 and AR (*r* = 0.021, whole cohort. *p* = 0.592). *p* values were calculated using two-tailed one-sample *z*-test for a correlation coefficient.

To ascertain the clinical significance of our findings, we investigated the correlation between MTA1 and PTEN in human prostate cancer by analyzing the expression of MTA1 and PTEN mRNA in the GSE41967 dataset [38] from the Gene Expression Omnibus (GEO) database. We observed a significant inverse correlation between MTA1 and PTEN that became stronger with severity of the disease as evidenced by the increased Gleason scores (Figure 3A). Moreover, as expected, we found a significant positive correlation between MTA1 and AKT1 (Figure 3B, top) and no correlation between MTA1 and AR (Figure 3B, bottom) in the same dataset.

Collectively, these data support a central role of MTA1 and MTA1-dependent signaling, including novel MTA1-Akt and MTA1-c-Myc feed-forward loops (Figure 2F), as significant drivers of *Pten*-loss induced prostate tumorigenesis and cancer progression suggesting potential benefits of MTA1-targeting approaches.

### Pterostilbene diminishes prostate cancer initiation, growth and progression in prostate-specific *Pten* loss mouse models

In chemoprevention modality, *Pten*^+/f^ mice were fed phytoestrogen-free AIN 76A diet supplemented with pterostilbene (100 mg/kg diet) for 8–10 months while in intervention modality *Pten* ^f/f^ mice were treated with daily pterostilbene (10 mg/kg bw) i.p. injections and monitored for prostate lesions at 6, 10, 15, 20, 25 and 33 weeks of age.

**Figure 4:**
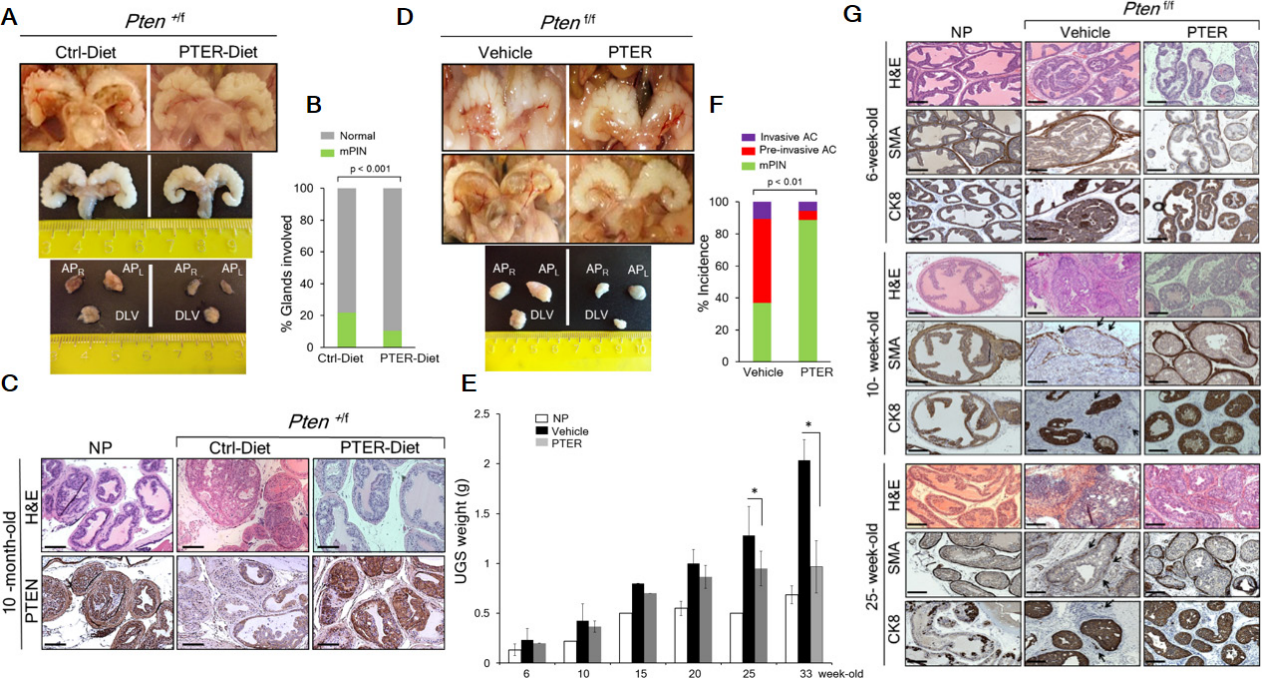
Pterostilbene reduces PIN formation in *Pten*^+/f^ and blocks progression to adenocarcinoma in *Pten*^f/f^ mice (**A**) Gross anatomy (top) and *ex vivo* images (middle) of urogenital system (UGS) and dissected prostate lobes (AP_R_, right anterior; AP_L_, left anterior, and DLV, dorso-latero-ventral) (bottom) of the representative prostates from 10-month-old *Pten*^+/f^ mice on phytoestrogen free AIN76 diet (Ctrl-Diet) and 100 mg/kg diet supplementation with pterostilbene (PTER-Diet). (**B**) Percentage of prostate glands from 10-month-old *Pten*^+/f^ mice on Ctrl− (*n* = 6) and PTER-Diet (*n* = 7) involved in high grade mouse PIN (mPIN). *p* < 0.001 (Fisher's exact test). (**C**) Comparison of H & E prostate histology and PTEN staining in representative 10-month-old mice with NP and *Pten*^+/f^ mice on Ctrl− and PTER-Diet. Scale bars, 100 μm. (**D**) Gross anatomy of the representative UGS from 10-week-old (top) and 33-week-old (middle) *Pten*^f/f^ mice treated with vehicle (DMSO) and 10 mg/kg bw PTER. Representative images of dissected prostate lobes of *Pten*^f/f^ mice (bottom). (**E**) Comparison of UGS weights of vehicle or PTER treated *Pten*^f/f^ mice, isolated at the indicated ages (*n* = 3/group). *p < 0.05 (two-tailed, two-sample *t*-test). (**F**) Incidence of mPIN, pre-invasive and invasive adenocarcinoma (AC) in *Pten*^f/f^ mice treated with vehicle (*n* = 19) and PTER (*n* = 18). *p* < 0.01 (Fisher's exact test). (**G**) Comparison of H & E, smooth muscle actin (SMA) and cytokeratin 8 (CK8) staining from representative 6-, 10- and 25-week-old mice with NP and vehicle or PTER treated *Pten*^f/f^ mice. Arrows indicate loss of SMA staining and CK8 positive luminal cells in the stroma of vehicle treated *Pten*^f/f^ mice as signs of invasiveness. Scale bars, 100 μm.

Gross anatomy (Figure 4A, top) and *ex vivo* images (Figure 4A, middle) of urogenital system (UGS) as well as dissected prostatic lobes (Figure 4A, bottom) from mice on PTER-Diet clearly indicated smaller prostates relative to controls while differences in food intake were not significant and the pterostilbene supplemented diet did not have any adverse effects on the mice (Supplementary Figure S1). All mice by 8–10 months of age developed high grade PIN, however mice on PTER-diet showed 50% reduction in the number of glands involved in PIN (Figure 4B and Supplementary Table S2) and more favorable histopathology with restored normal ductal structures accompanied by higher PTEN protein expression, as evident by H & E and PTEN staining (Figure 4C).

In the *Pten*-null model, by 10 weeks of age, 67% of the *Pten*^f/f^ mice contained regions of pre-invasive adenocarcinoma with enlarged, hardened prostates, which progressed to invasive adenocarcinoma by 25–33 weeks of age in all the mice examined but shrank upon pterostilbene treatment as evident by gross anatomy (Figure 4D, top and middle), *ex vivo* images of dissected prostatic lobes (Figure 4D, bottom), and UGS weights (Figure 4E). Overall, 64% of the vehicle-treated mice exhibited pre-invasive or invasive adenocarcinoma whereas daily 10 mg/kg pterostilbene treatment reduced the incidence of adenocarcinomas to 12% and halted the progression at PIN stage (Figure 4F and 4G and Supplementary Table S3). As seen in Figure 4G and Supplementary Figure S2, vehicle-treated mice developed enlarged prostates characterized by disorganized glandular structures, which contained regions of cribriform carcinoma with signs of microinvasion (loss of the basal layer (SMA) and CK8-positive luminal cells that escaped into reactive stroma). In contrast, PTER-treated mice mostly showed characteristics of PIN, which retained a basal layer of SMA-positive cells and CK8-positive luminal cells along with residual hypercellularity. We did not find any lymph node metastasis even in mice aged over one year, by examining in total 34 renal and iliac lymph nodes of control or treated mice (Supplementary Figure S3).

To determine whether pterostilbene reaches the target tissue, we analyzed pterostilbene concentrations in prostate tissues, as well as in the serum, from *Pten*^+/f^ and *Pten*^f/f^ mice. We found accumulation of pterostilbene in the prostates from both mouse models, with apparent greater accumulation when administered i.p. (Supplementary Table S4), suggesting that high lipophilicity, membrane permeability and metabolic stability of pterostilbene [19–21] secure its potent biological activity *in vivo*.

### Pterostilbene targets *Pten* loss-induced MTA1 upregulation and its associated signaling

**Figure 5:**
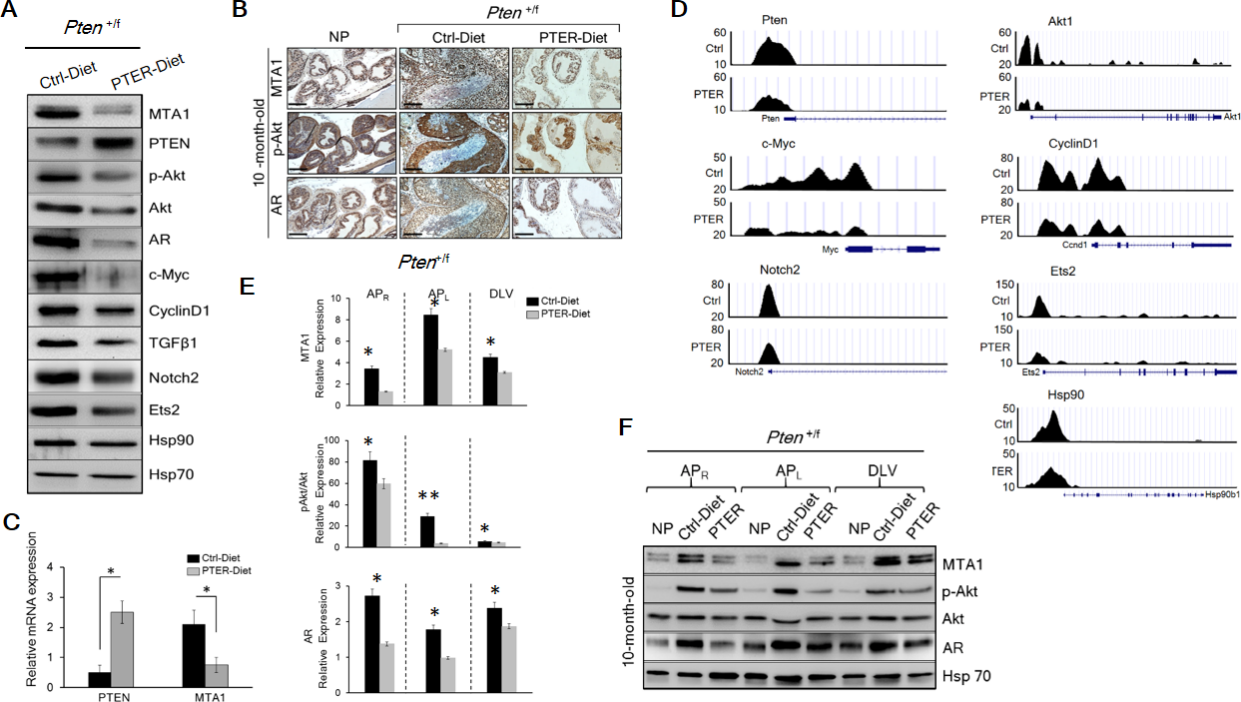
Inhibition of MTA1 and its associated signaling by pterostilbene (PTER) in *Pten*^+/f^ mice (**A**) Immunoblots of MTA1, PTEN, p-Akt, Akt, AR, c-Myc, CyclinD1, TGFβ1, Notch2, Ets2, and Hsp90 of prostate tissues from representative 10-month-old *Pten*^+/f^ mice on Ctrl- and PTER-Diet. Hsp70 was a loading control. (**B**) Comparison of MTA1, p-Akt and AR IHC staining of the prostate sections from representative 10-month-old *Pten*^+/f^ mice on Ctrl- and PTER-Diet. NP, normal prostate. Scale bars, 100 μm. (**C**) qRT-PCR of PTEN and MTA1 mRNA levels in prostate tissues from 10-month-old *Pten*^+/f^ mice on Ctrl- and PTER-Diet. Data are mean ± SEM (*n* = 3), *p < 0.05; **p < 0.01 (two-tailed, two-sample *t*-test). (**D**) Comparative analysis of MTA1 binding in the prostate tissues of *Pten*^+/f^ mice on Ctrl- and PTER-Diet. Representative MTA1 ChIP-Seq tracks for Pten, Akt1, c-Myc, CyclinD1, Notch2, Ets2 and Hsp90 gene loci at 10 kb resolution are shown. (**E**) Quantitation of MTA1, p-Akt/Akt and AR expression in prostate lobes of 10 month-old *Pten*^+/f^ mice on Ctrl- and PTER-Diet (see F). Data represent the mean ± SEM (*n* = 3), *p < 0.05; **p < 0.01 (two-tailed, two-sample *t*-test). (**F**) Immunoblots of MTA1, p-Akt, Akt and AR in the dissected prostatic lobes (AP_R_, right anterior; AP_L_, left anterior, and DLV, dorso-latero-ventral) from 10-month-old *Pten*^+/f^ mice on Ctrl- and PTER-Diet. Hsp70 was used as a loading control.

To elucidate the molecular mechanisms responsible for pterostilbene efficacy, we examined its effects on MTA1 expression in prostate tissues from *Pten*^+/f^ and *Pten*^f/f^ mice. We found downregulation of MTA1 protein levels in *Pten*^+/f^ prostates (Figure 5A and 5B). Remarkably, these prostate tissues showed elevated levels of PTEN protein (Figure 5A and Supplementary Figure S4C), highlighting the potential of dietary epigenetic therapy to restore the expression as well as the activity of the remaining PTEN allele, which can be further gauged by decreased p-Akt levels (Figure 5A and 5B). Additionally, PTEN and MTA1 gene expression analysis by qRT-PCR demonstrated elevated PTEN and decreased MTA1 mRNA levels in the prostate tissues from mice on PTER-diet (Figure 5C), suggesting transcriptional regulation of MTA1 by pterostilbene with consequent decreased MTA1 occupancy of PTEN promoter (Figure 5D and Supplementary Table S1). This is consistent with known MTA1-PTEN relationship [17, 39].

Since there is a direct relationship between MTA1 and Akt (Figure 1B and 1C and Figure 2A and 2B), pterostilbene prevents the activation of Akt pathway, at least in part, by targeting MTA1 and thereby decreasing MTA1 occupancy of Akt1 promoter (Figure 5D). Moreover, pterostilbene reverses the MTA1-associated perturbation of key oncogenes as demonstrated by the decreased levels of c-Myc, CyclinD1, Notch2, Ets2 and Hsp90 proteins in the prostate tissues (Figure 4A) through decreased MTA1 occupancy of these target promoters (Figure 5D and Supplementary Table S1). In addition, prostates from mice on PTER-Diet exhibited downregulation of TGFβ1, a well-known upstream activator of MTA1 [40] (Figure 4A). Interestingly, although independent from MTA1, pterostilbene also downregulated the AR levels (Figure 5A and 5B), further strengthening its potential as a suitable chemopreventive agent to reduce the risk of prostate cancer.

To further investigate the lobe-specific differential impact of pterostilbene treatment, we performed immunoblot analysis using right anterior (AP_R_), left anterior (AP_L_) and dorso-latero-ventral (DLV) prostatic lobes from *Pten*^+/f^ mice on control and PTER diet along with Cre-negative (NP) mice (Figure 5E and 5F). In normal prostates, MTA1 was detected at very low levels in the lobes. In contrast, MTA1 was highly expressed in all lobes of *Pten*^+/f^ with various intensities, the highest being detected in AP_L_. Importantly, the increase in MTA1 levels in all lobes in *Pten* heterozygous mice was profoundly inhibited by pterostilbene (Figure 5E and 5F). Pterostilbene treatment also inhibited p-Akt and AR levels, with the effects most evident in AP.

**Figure 6:**
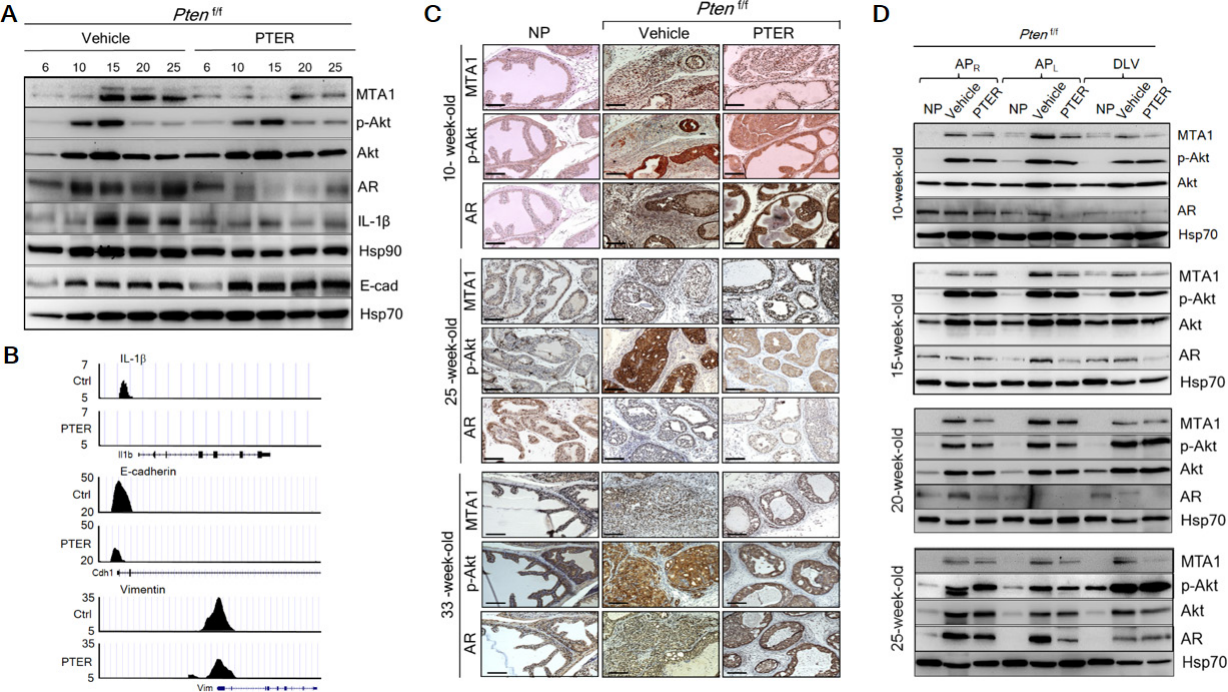
Inhibition of MTA1 and its associated signaling by pterostilbene (PTER) in *Pten*^f/f^ mice (**A**) Immunoblots of MTA1, p-Akt, Akt, AR, IL-1β, Hsp90 and E-cadherin (E-cad) in the prostate tissues of vehicle and PTER treated *Pten*^f/f^ mice, isolated at indicated ages. Hsp70 was a loading control. (**B**) Comparative analysis of MTA1 binding in the prostate tissues of *Pten*^+/f^ mice on Ctrl- and PTER-Diet. Representative MTA1 ChIP-Seq tracks for IL-1β, E-cadherin and Vimentin gene loci at 10 kb resolution are shown. (**C**) Comparison of IHC staining of MTA1, p-Akt and AR in the prostate sections with carcinoma lesions from representative 10-, 25- and 33-week old vehicle or PTER treated *Pten*^f/f^ mice and NP controls. Scale bars, 100 μm. (**D**) Immunoblots of MTA1, p-Akt, Akt and AR in the dissected prostate lobes from vehicle or PTER-treated 10-, 15-, 20- and 25-week-old *Pten*^f/f^ mice. Hsp70 was used as a loading control. For quantitation of MTA1, p-Akt/Akt and AR expression in prostate lobes of *Pten*^f/f^ mice at different ages (*n* = 3/group) see Supplementary Figure S5.

In the intervention strategy with cancer-prone *Pten*-null mice, in which progressive increase in MTA1 levels were associated with age-related aggressiveness of prostate cancer (Figure 1E), we found a substantial decrease in MTA1 levels in response to pterostilbene treatment at any ages examined by immunoblotting or IHC (Figure 6A and 6C and Supplementary Figure S4). Moreover, the downregulation of MTA1 by pterostilbene significantly altered the MTA1-dependent expression of pro-inflammatory IL-1β and Hsp90, and E-cadherin (Figure 6A), possibly through decreased MTA1 occupancy of these gene promoters (Figure 6B). Importantly, pterostilbene intervention inhibited MTA1-associated maintenance of already highly activated Akt pathway (Figure 6A and Supplementary Figure S5). Here again, pterostilbene treatment showed downregulation of AR levels (Figure 6A and 6C). In the context of prostate lobes, once again, MTA1 was highly expressed in all lobes of *Pten*^f/f^ prostates with various intensities, and the highest fold increase, compared to normal prostates, was observed in the anterior lobes (Figure 6D and Supplementary Figure S5). Age and progression related increases in MTA1 were inhibited on an average by 50% in all prostatic lobes of mice treated with pterostilbene (Figure 6D and Supplementary Figure S5). Pterostilbene had less consistent p-Akt-inhibitory effects in different lobes; yet, remarkably the effect was mostly evident in the AP_L_, in which MTA1 inhibition by pterostilbene was most significant. It is quite possible that one of the mechanisms of pterostilbene inhibition of the Akt pathway in these mice is through MTA1. Finally, AR levels were also markedly reduced in the prostates from the pterostilbene-treated group compared to control mice (Figure 6D and Supplementary Figure S5).

Altogether, diet with pterostilbene supplementation or daily pterostilbene injections in *Pten*^+/f^ and *Pten*^f/f^ immunocompetent pre-clinical mouse models, respectively, reduced the profoundly elevated MTA1 levels and inhibited the coordinate expression of multiple components of MTA1 tumor-promoting signaling, demonstrating the *in vivo* efficacy of pterostilbene as a MTA1-targeted chemopreventive and intervention strategy.

### Pterostilbene reduces MTA1-induced cellular proliferation and angiogenesis, and promotes MTA1-dependent apoptosis in *Pten* loss-driven prostate cancer

**Figure 7:**
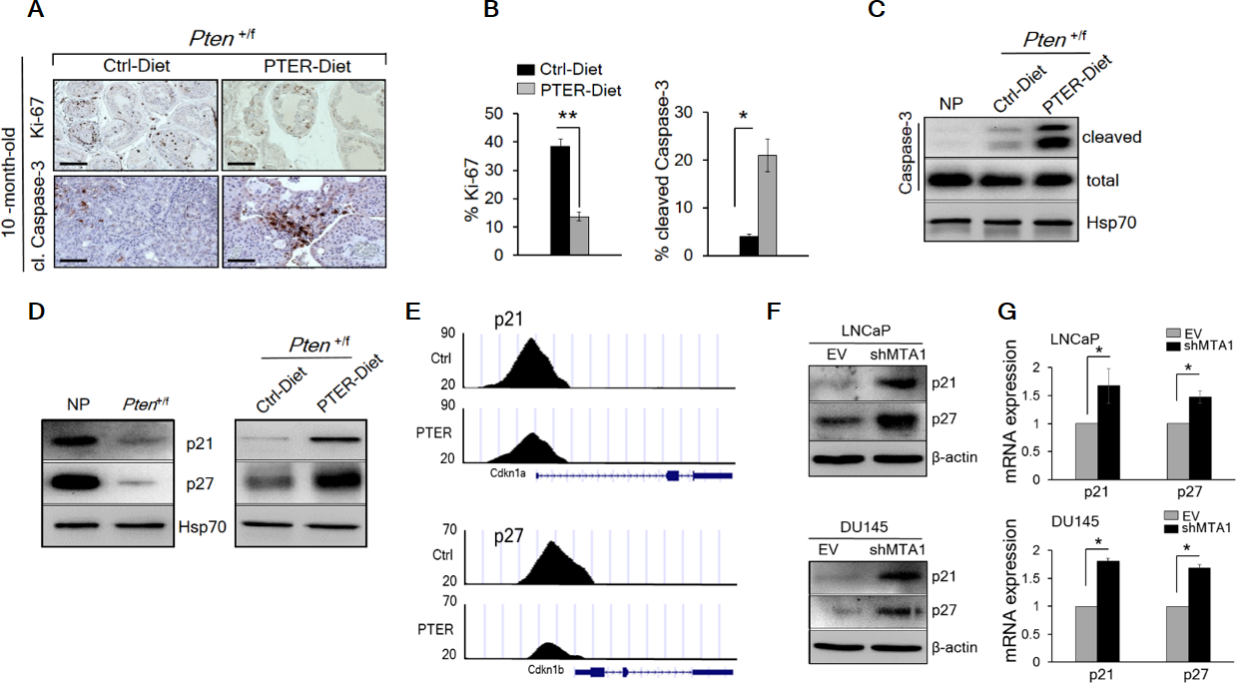
Pterostilbene significantly inhibits MTA1-dependent cell proliferation and induces MTA1-targeted apoptosis in *Pten*^+/f^ mice (**A**) Representative Ki-67 (top) and cleaved Caspase-3 (bottom) staining of prostate tissues from 10-month-old *Pten*^+/f^ mice on Ctrl- and PTER-Diet. Scale bars, 100 μm (Ki-67) and 50 μm (cleaved Caspase-3). (**B**) Quantitation of Ki-67 (left) and cleaved Caspase-3 (right) positive cells of prostate tissues from 10-month-old *Pten*^+/f^ mice on Ctrl- and PTER-Diet (*n* = 5/group). (**C**) Immunoblots of total and cleaved Caspase-3 in prostate tissues of 10-month-old *Pten*^+/f^ mice on Ctrl- and PTER-Diet. (**D**) Immunoblots of p21 and p27 in the prostate tissues of 10-month-old *Pten*^+/f^ mice compared to NP controls (left) and mice on Ctrl- and PTER-Diet (right). (**E**) Comparative analysis of MTA1 binding in the prostate tissues of *Pten*^+/f^ mice on Ctrl- and PTER-Diet. Representative ChIP-Seq tracks for p21 and p27 gene loci at 10 kb resolution are shown. (**F**) Immunoblots of p21 and p27 in LNCaP (top) and DU145 (bottom) cells expressing (EV) and silenced for MTA1 (shMTA1). (**G**) qRT-PCR of p21 and p27 mRNA levels in cells expressing MTA1 and silenced for MTA1 (shMTA1). Data are mean ± SEM (*n* = 3), *p < 0.05; **p < 0.01 (two-tailed, two-sample *t*-test).

**Figure 8:**
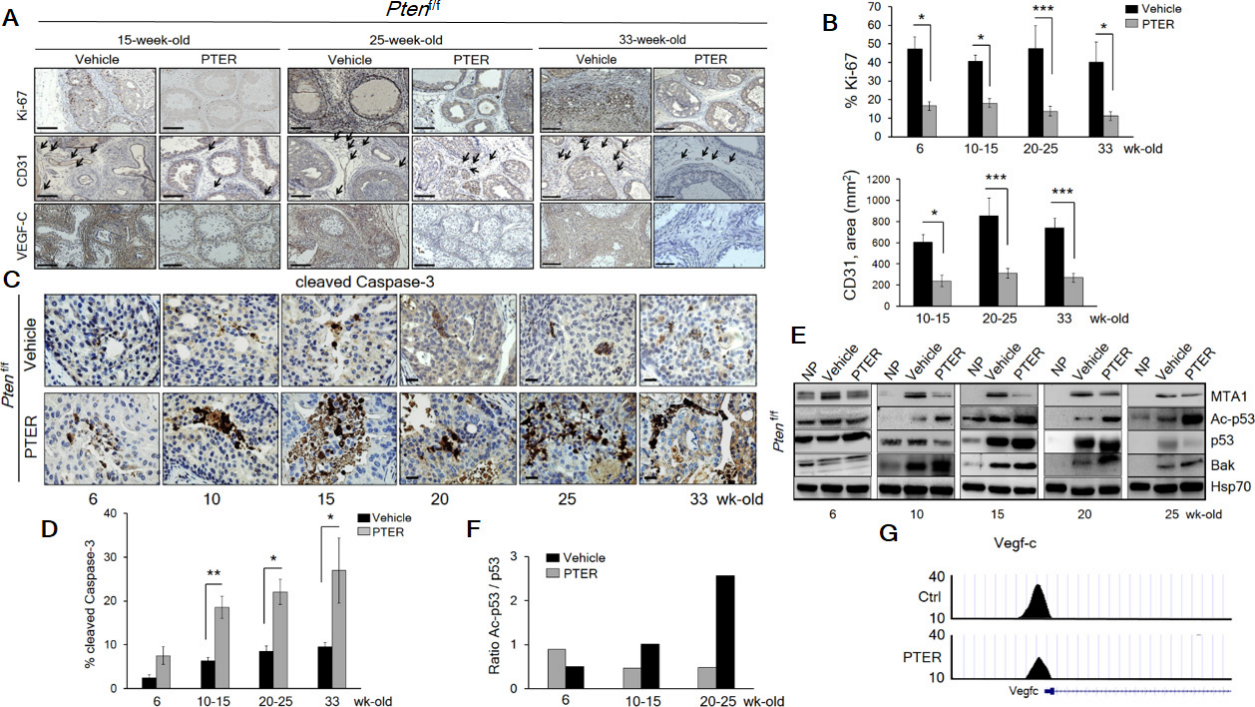
Pterostilbene significantly inhibits MTA1-dependent cell proliferation and angiogenesis and induces MTA1-targeted apoptosis in *Pten*^f/f^ mice (**A**) Representative Ki-67 (top, each panel), CD31 (middle, each panel) and VEGF-C (bottom, each panel) staining of the prostate tissues from *Pten*^f/f^ mice treated with vehicle and PTER, at indicated ages. Arrows indicate vessels. Scale bars, 100 μm. (**B**) Quantitation of Ki-67 (top) and CD31 (bottom) positive cells of prostate tissues from mice treated with vehicle and PTER (*n* = 5/group). (**C**) Representative images and (**D**) Quantitation of cleaved Caspase-3 staining at the indicated ages of vehicle and PTER treated *Pten*^f/f^ mice (*n* = 5/group). Scale bars, 10 μm. Data are mean ± SEM (*n* = 3), *p < 0.05; **p < 0.01; ***p < 0.001 (two-tailed, two-sample *t*-test). (**E**) Immunoblots of MTA1, Ac-p53, p53 and Bak in the prostate tissues from vehicle and PTER treated *Pten*^f/f^ mice, isolated at the indicated ages. NP, normal prostate. Hsp70 was used as loading controls from prostate tissues. (**F**) Densitometry of the Ac-p53/p53 ratio from the representative blot. (**G**) Comparative analysis of MTA1 binding in the prostate tissues of *Pten*^+/f^ mice on Ctrl- and PTER-Diet. Representative MTA1 ChIP-Seq tracks for Vegf-c gene locus at 10 kb resolution are shown.

The restoration of a more favorable histopathology by pterostilbene in both *Pten*^+/f^ and *Pten*^f/f^ mice through MTA1 targeting and its associated signaling may functionally involve inhibition of proliferation and induction of apoptosis. The number of Ki-67 positive tumor cells was decreased by about three fold in mice on pterostilbene diet (Figure 7A, top and 7B, left) and about three to five fold in mice of different ages injected with pterostilbene (Figure 8A and 8B, top and Supplementary Figure S6). Moreover, immunostaining and immunoblot analyses for cleaved Caspase-3 showed that apoptosis in prostate tissues from mice on pterostilbene diet was substantially increased (four to five-fold) (Figure 7A, bottom and 7B, right and 7C). Notably, pterostilbene treatment in the *Pten*^f/f^ mice also exhibited a marked and an age-dependent increase in cleaved Caspase-3 (Figure 8C and 8D), indicative of prolonged treatment benefits.

Since we found p21 and p27, known key regulators of proliferation and apoptosis [41–43] among the targets identified from MTA1 ChIP-Seq analysis (Supplementary Table S1), we demonstrated low levels of p21 and p27 in *Pten*^+/f^ prostate tissues (Figure 7D, left), which was rescued by pterostilbene diet (Figure 7D, right) due to a decreased occupancy of MTA1 onto p21 and p27 promoter (Figure 7E). Further, we validated the upregulation of these molecules at protein and mRNA levels in MTA1 knockdown human prostate cancer cells (Figure 7F and 7G).

We previously reported that resveratrol/pterostilbene suppressed the MTA1-dependent decrease in acetylation of p53, which led to decreased apoptosis in prostate cancer [13, 15]. In *Pten*^f/f^ prostates, we found age-dependent increased p53 acetylation together with induction of pro-apoptotic Bak upon pterostilbene treatment (Figure 8E and 8F).

Finally, consistent with our previous reports on the link between MTA1 and angiogenesis [9, 13] and our current finding of VEGF-C as a MTA1 target (Supplementary Table S1), pterostilbene treatment led to decreased hemangiogenesis and lymhangiogenesis, as evident by CD31 and VEGF-C immunostaining (Figure 8A, middle and bottom and 8B, bottom and Supplementary Figure S6). In addition to the already observed direct link between MTA1 and the pro-angiogenic factor IL-1β [44] (Figure 1E and Figure 2A) and its reversal by pterostilbene treatment (Figure 6A), ChIP-Seq analysis showed decreased MTA1 occupancy of VEGF-C promoter upon pterostilbene treatment (Figure 8G). Taken together, these results demonstrate that pterostilbene treatment inhibits tumor cell proliferation and angiogenesis and induces apoptosis in *Pten*-loss prostate tumors, at least in part, due to inhibition of MTA1.

## DISCUSSION

**Figure 9:**
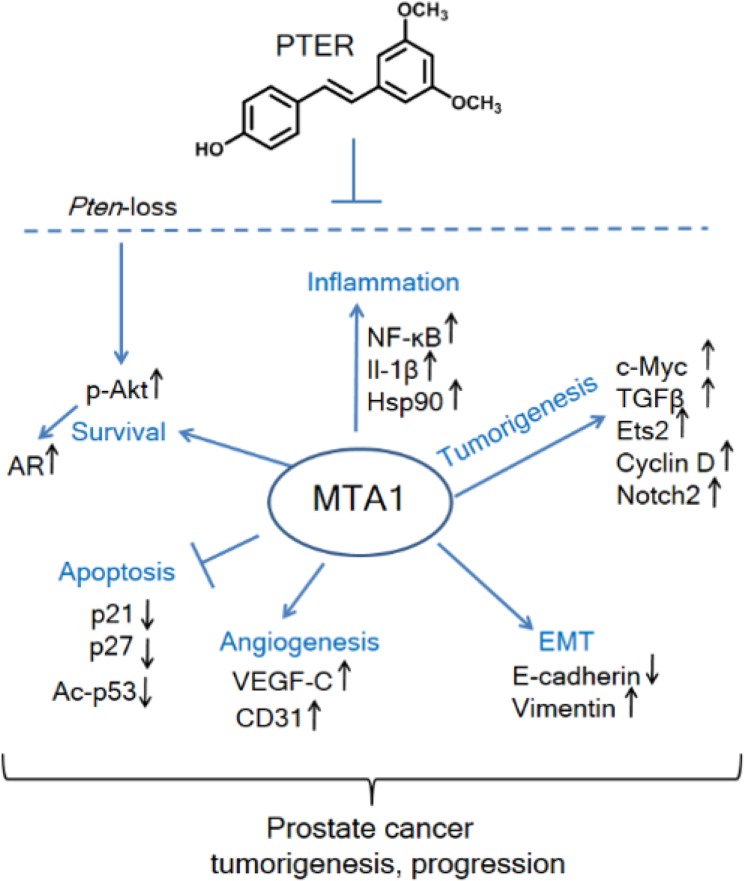
Schematic representation of MTA1-targeted effects of pterostilbene in prostate cancer Significantly increased levels of MTA1, a key upstream epigenetic regulator, promote inflammation, tumorigenesis, EMT, angiogenesis, and survival signaling and repress apoptosis. Pterostilbene (PTER) targets MTA1 and MTA1-guided molecular drivers of tumor promotion, thereby blocking the *Pten* loss-driven prostate tumorigenesis and cancer progression.

Here, we report on a previously unknown mechanism-based importance of the chromatin modifier MTA1 in *Pten* loss-driven prostate tumors. Mechanistically, we demonstrated that aberrant overexpression of MTA1 resulted in activation of MTA1-dependent transcriptional signatures that promote proliferation, inflammation, invasion and survival of cancer cells (Figure 9). Importantly, we demonstrated that pharmacological inhibition of MTA1 and its associated network by a natural dietary compound pterostilbene, exhibited chemopreventive and therapeutic efficacy as evident by the decreased severity of PIN and prevention of progression to carcinoma in pre-clinical *Pten*-loss mouse models of prostate cancer.

Our data appear highly clinically relevant because they support the concept that patients with deregulated PTEN/Akt pathway, which comprise 21–42% of prostate cancer patients [45], most likely have overexpression of MTA1 and may benefit from MTA1-targeted therapy. Moreover, since increased levels of MTA1 is associated with increased Akt activity and PI3-kinase inhibitors also target MTA1 (Figure 2D), combinatorial therapy using pterostilbene and PI3-kinase inhibitors may be considerably more effective.

Results from our ChIP-Seq analysis identified oncogenic c-Myc, Cyclin D1, Ets2, Akt1 and Notch2, pro-inflammatory Hsp90, pro-lymphangiogenic VEGF-C, and pro-apoptotic p27 as novel transcriptional targets of MTA1, revealing its ability to simultaneously activate multiple tumor-promoting pathways. The findings of the direct link between MTA1 and pro-lymphangiogenic VEGF-C, which promotes tumor metastasis to distant organs [46, 47], one more time emphasizes the importance of MTA1 in the promotion of prostate cancer metastasis to distant organs such as bone [9, 13]. Results from ChIP-Seq analysis define MTA1 as a key upstream epigenetic driver in prostate cancer initiation and progression. Notably, pterostilbene reduced MTA1 occupancy of all target promoters as evident by resulting lower binding peaks.

We previously reported on the ability of MTA1 to regulate activation of the PTEN/Akt pathway through deacetylation of PTEN [17]. Others have reported MTA1 transcriptional repression of PTEN [39]. Here, we confirmed the inverse relationship between MTA1 and PTEN on the transcriptional level, and also identified MTA1 binding to PTEN promoter by ChIP-Seq analysis. The rescue of PTEN mRNA and protein expression in *Pten*^+/f^ mice upon pterostilbene-mediated downregulation of MTA1 conveys the feasibility of therapeutically attractive approaches for PTEN re-activation by epigenetic-targeted agents.

One of the most striking observations in our models was that MTA1 levels were dramatically increased not only in prostate epithelial cells but also in reactive stroma. While there is a well-established association between inflammation, reactive stroma and prostate cancer [48], the underlying mechanisms remain elusive. Our study demonstrate the possible role of MTA1 in epithelial-stromal interaction. Importantly, since the oncogenic potential of MTA1 might also be linked to other cell types in the reactive stroma, the anti-inflammatory effects of pterostilbene mediated through MTA1-associated NF-ĸB, IL-1β, Hsp90 may play an important additive beneficiary role in its antitumor activity.

Although both MTA1 and AR had similar patterns of behavior, we did not find any direct evidence indicating that overexpression of MTA1 could contribute to the activation of AR signaling. Importantly, the inhibitory effects of pterostilbene on AR levels in both luminal cells and reactive stroma represent additional beneficiary factor, which potentially defines favorable histopathology of the lesions upon pterostilbene exposure. Our studies in human prostate cancer cells also demonstrated inhibitory effects of pterostilbene on AR levels [49]. This data reason that combined inhibition of MTA1 and AR by pterostilbene provides for its maximal efficacy in *Pten* deficient tumors.

The concept of “epigenetic therapy” for cancer has developed during the last two decades [50, 51], and efforts were largely driven towards the development of inhibitors of druggable epigenetic enzymes such as inhibitors of DNA methylation or histone deacetylation [50]. Limited studies are available on targeting other epigenetic regulators, such as epigenetic readers and transcription factors [52]. Our serendipitous finding of MTA1 as a new molecular target of resveratrol and its analogs [13, 15] opened opportunities for further pre-clinical validation of the efficacy of pterostilbene as natural epigenetic therapy in prostate cancer. Others have also demonstrated anticancer effects of pterostilbene in prostate cancer through multiple mechanisms, which include reduction of prostate-specific antigen, promotion of apoptosis, alteration of cell cycle and inhibition of cell growth [53–55]. While the improved bioavailability of pterostilbene and its distribution in various tissues has been reported [19–22], we, for the first time, detected accumulation of pterostilbene in the prostate tissues, providing evidence that pterostilbene reaches the target organ. This finding may be clinically relevant since the dosage we used both in diet and i.p. was roughly equivalent to the reported non-toxic and effective doses in rodents [20, 56, 57] and human [58, 59].

In summary, using clinically relevant disease models of prostate cancer, we demonstrated the MTA1-targeted chemopreventive and therapeutic efficacy of pterostilbene. We also identified panel of MTA1-guided molecules that are responsive to pterostilbene treatment and, therefore, can be utilized not only as prognostic but also as predictive biomarkers, with some of them having the feasibility of being detected in the blood (IL-1β, VEGF). Our findings offer a solid foundation to endorse chemopreventive and interventional clinical trials with pterostilbene for personalized targeted prostate cancer management. Thus, it is likely that targeting MTA1 and MTA1-associated molecular and cellular events by dietary pterostilbene in the high-risk population and patients with early stages of prostate cancer, i.e. patients on active surveillance with deregulated MTA1, could be the most needed immediate chemopreventive strategy. Moreover, in the future, as other pharmacological inhibitors of MTA1 such as HDAC inhibitors [15] or PI3-kinase inhibitors (Figure 2D) are validated *in vivo*, combinatorial strategies with pterostilbene can be considered for more effective but less toxic therapeutic approaches in targeted patient population with advanced disease.

## MATERIALS AND METHODS

### Reagents

Phytoestrogen-free AIN 76A diet was obtained from Research Diets, Inc. Pterostilbene was synthesized according to protocol described previously [60]. The structure was confirmed by spectroscopy. The purity of pterostilbene was determined to be > 99. 9%. Pterostilbene powder was shipped to Research Diets, Inc for formulation of pterostilbene supplemented AIN 76A diet at a concentration of 100 mg/kg diet. Upon receiving pterostilbene supplemented diet, it was aliquoted into separate portions, sealed in aluminum foil and kept at 4°C until use. For injections, pterostilbene (10 mg/kg bw) was suspended in 10% DMSO, freshly every day and kept in dark until use.

### Cell culture

Human prostate cancer cells, LNCaP, DU145 (ATCC) and PC3M (a gift from Dr. Bergman, Northwestern University) were cultured in RPMI1640 (Life Technologies) containing 10% FBS and antibiotics at 37°C and 5% CO_2_. Establishment of prostate cancer cell lines with stable MTA1 knockdown (MTA1shRNA) has been described and characterized previously [13, 15]. All cell lines were last authenticated using short tandem repeat profiling at Research Technology Support Facility, Michigan State University in 2014. Cells were tested for mycoplasma using the Universal Mycoplasma Detection Kit (ATCC).

### Generation of prostate-specific *Pten* heterozygous and knockout mice

Animal housing, care and treatments were in accordance with approved protocol #1272A by Institutional Animal Care and Use Committee of UMMC. During the study, animals were permitted free access to drinking water and food, and were monitored daily for their general health. C57BL/6J mouse homozygous for the “floxed” allele of *Pten* gene (*Pten* ^f/f^) was purchased from Jackson Laboratories and bred with *Pb-Cre4* male from the B6.Cg genetic background (NCI mouse repository) that specifically express *Cre* recombinase in the prostate epithelium [23]. Tail-genotyping was performed using the following primers: PTEN geno olMR9554F:5′-CAA GCA CTC TGC GAA CTG AG-3′; PTEN geno olMR9555R:5′-AAG TTT TTG AAG GCA AGA TGC-3′ with wt band of 156 bp and mutant band of 328 bp; and Cre F: 5′-TCG CGA TTA TCT TCT ATA TCT TCA G-3′; Cre R: 5′-GCT CGA CCA GTT TAG TTA CCC-3′ with a band of 393 bp. PCR was performed on an Eppendorf thermocycler. We used male *Pb-Cre4*; *Pten*^+/f^ prostate-specific heterozygous mice (abbreviated as *Pten*^+/f^ in the text and figures) in experiments with dietary supplementation of pterostilbene, and male *Pb-Cre4*; *Pten*^f/f^ prostate-specific knockout mice (abbreviated as *Pten*^f/f^ in the text and figures) in experiments with i.p. injections of pterostilbene. Normal prostates (NP) from *Cre*-negative; *Pten*^+/f^ or *Cre*-negative; *Pten*^f/f^ were processed as normal controls.

### Dose calculations

In our previous study with orthotopic prostate cancer xenografts, we extrapolated the dose from resveratrol chemopreventive studies in human colorectal cancer [61] by using Body Surface Are (BSA) formula for dose translation [62] and demonstrated that both resveratrol and pterostilbene had anticancer and antimetastatic effects at 50 mg/kg/day, i.p. administration, with higher accumulation of pterostilbene in serum. Bearing in mind known higher bioavailability of pterostilbene and lower weight of pups used in the current study, we finalized pterostilbene dose at 10 mg/kg bw per day, i.p. for this study. In addition, we accumulated circumstantial data with higher doses of pterostilbene (25, 50 and 100 mg/kg bw). For the calculation of concentration of pterostilbene in the diet, we used DD = (SD × BW)/FI formula (Research Diets, Inc) where, DD is Diet Dose (mg compound/kg Diet); SD is Single Dose (mg compound/kg bw/day); BW is Body Weight (g bw/animal) and FI is Daily Food Intake (g Diet/day). We finalized pterosilbene dose at 100 mg/kg diet.

### Treatment

After series of carefully designed breeding strategies and genotyping, we collected *Pten*^+/f^ male mice, which after simple randomization, were maintained on AIN 76A (*n* = 21) and AIN 76A diet supplemented with pterostilbene (*n* = 30) until sacrifice (chemoprevention design). For an intervention study we procured a total of 64 *Pten*^f/f^ male mice maintained on AIN76A diet and employed by simple randomization with slightly larger allocation probability for treatment group. Mice were treated five days per week i.p with either 10% DMSO (vehicle control, *n* =31) or 10 mg/kg bw of pterostilbene (*n* = 33). The animals were started on treatment immediately after weaning for either 3 weeks (6 week-old), 7 weeks (10 week-old), 12 weeks (15 week-old), 17 weeks (20 week-old), 22 weeks (25 week-old) or 30 weeks (33 week-old). As an overall control, an additional group of littermates with *Cre*- negative *Pten*^+/f^ or *Pten*^f/f^ genotype, which possess normal prostates were also put on AIN 76A diet. Necropsy was performed at 8–10 months for *Pten*^+/f^ and respective time points (3–30 weeks of treatment) for *Pten*^f/f^ mice.

### Mouse procedures

At the time of sacrifice, an abdominal midline incision was made and lower urogenital tract, including prostate, seminal vesicles and bladder was removed *en bloc* and washed with cold PBS. Dissection of different lobes of each prostate [left anterior prostate (AP_L_); right AP (AP_R_) and the entire dorso-latero-ventral (DLV) lobe] was done under dissecting microscope. Tissues were homogenized, and protein lysates were made using RIPA buffer. For histological analysis the urogenital system was fixed with 10% neutral-buffered formalin (see below). For visualization and isolation of lymph nodes, 15-week-old mice and older were anesthetized with 2.0% isoflurane, and 5% Evans Blue dye (Sigma-Aldrich) was injected s.c. into the mouse hind footpad [63]. After 30 min, mice were euthanized with CO_2_ and dissected to locate lymph nodes of interest. The blue-labeled iliac and renal lymph nodes were removed, washed in PBS and fixed with 10% neutral-buffered formalin. To check for gross metastasis, a laparotomy was performed. Necropsy notes were collected for all animals with descriptions of the prostate, seminal vesicles, bladder and other organs or tissues showing any abnormalities. In addition, photographs were made of gross anatomy of mice urogenital systems. At the end, blood was collected and serum was obtained by centrifugation and stored at −80°C.

### Histopathology and immunohistochemistry

Tissue paraffin embedding, sectioning and H & E staining were performed by the Histology Core facility, Department of Pathology, UMMC. Sections (4 μm) were prepared from formalin-fixed paraffin embedded tissues and mounted on slides. Histological sections were prepared by hematoxylin and eosin (H & E) staining and were evaluated independently by two pathologists (JML and JRL) who were blinded to the treatment. Immunohistochemistry was performed as described previously [9, 13] using antibodies against Ki-67, CK8, SMA, MTA1, pAkt, PTEN, AR, CD31; VEGF-C and cleaved caspase-3 (see “Antibodies” section), the Vectastain ABC Elite Kit and the ImmPACT DAB kit (Vector Laboratories). Images were viewed and recorded on Nikon Eclipse 80i microscope. The ImageTool software was used to count positively-stained cells in five randomly selected fields.

### RNA analysis

Mouse prostates and cell pellets were harvested and immediately stored in RNA later (Sigma-Aldrich) at −80°C until analysis. Prostate tissues were homogenized, after which total RNA fraction was isolated using the RNeasy mini kit (Qiagen). The quality of the RNA was determined on a Bio-Rad Experion analyzer. PCR was performed on a CFX96 Real Time PCR Detection System (Bio-Rad) using the primer sequences given in Supplementary Table S5, and fold changes in gene expression was determined using the 2^−ΔΔCt^ method [64].

### Immunoblot analysis

Lysates were prepared from homogenized prostate tissues and the cell lines in the RIPA buffer containing protease and phosphatase inhibitor cocktail (ThermoFisher Scientific). 70 μg of protein was loaded in 10–15% SDS-PAGE and transferred onto polyvinylidene difluoride (PVDF) membranes. Membranes were incubated in 5% nonfat dry milk/TBST blocking buffer for 1 h at room temperature, followed by an overnight incubation at 4°C in the presence of corresponding antibodies (see “Antibodies” section). Membranes were washed with TBST and incubated in the presence of HRP-conjugated secondary antibodies. Signal detection was carried out using SuperSignal West Dura chemiluminescent substrate (ThermoFisher Scientific). Signal quantitation was conducted with Image J software (http://rsb.info.nih/gov/nih-image/).

### Antibodies

Antibodies to the following markers were used for IHC and Western blots: rabbit MTA1 (D40D1) (Cell Signaling Technologies, #5647, 1:50 for IHC, 1:1000 for western blotting); rabbit p-Akt (D9E) (Cell Signaling Technologies, #4060, 1:50 for IHC, 1:1000 for western blotting); rabbit PTEN (D4.3) (Cell signaling Technologies, #9188, 1: 125 for IHC, 1:1000 for western blotting); rabbit AR (N-20) (Santa Cruz Biotechnologies, sc-816, 1:500 for IHC, 1:500 for western blotting); rabbit cleaved caspase-3 (D175)(5A1E) (Cell Signaling Technologies, #9664, 1:100 for IHC, 1:1000 for western blotting); rabbit Ki-67 (SP6) (Abcam, ab16667, 1:100 for IHC); rabbit SMA (Abcam, ab5694, 1:800 for IHC); rabbit CK8 (EP16284) (Abcam, ab53280, 1:800 for IHC), rabbit CD31 (SP-38) (Novus Biologicals, NBP1-49805, 1:500 for IHC); rabbit VEGF-C (Novus Biologicals, NB110-61022, 1:100 for IHC). The following antibodies were used for western blot analysis: rabbit Akt (C67E7) (Cell Signaling Technologies, #4691, 1:1000); rabbit Hsp70 (W27) (Santa Cruz Biotechnologies, sc-24, 1:1000); rabbit Hsp90 (H-114) (Santa Cruz Biotechnologies, sc-7947, 1:1000); rabbit Ac-p53 (Abcam, ab61241, 1:100); mouse p53 (3H2820) (Santa Cruz Biotechnologies, sc-71821, 1:100); mouse Bak (G-23) (Santa Cruz Biotechnologies, sc-832, 1:100); rabbit Vimentin (D21H3) (Cell Signaling Technologies, #5741, 1:1000), rabbit E-cadherin (24E10) (Cell Signaling Technologies, #3195, 1:1000); goat IL-1β (C-20) (Santa Cruz Biotechnologies, sc-1250, 1:200), rabbit NF-κB p65 (H-286) (Santa Cruz Biotechnologies, sc-7151, 1:200); rabbit p21 (C-19) (Santa Cruz Biotechnologies, sc-397, 1:100); mouse p27 (F-8) (Santa Cruz Biotechnologies, sc-1641, 1:100), mouse Ets2 (E-5) (Santa Cruz Biotechnologies, sc-365666, 1:200); rabbit TGF-β1 (V) (Santa Cruz Biotechnologies, sc-146, 1:200); rabbit c-Myc (N-262) (Santa Cruz Biotechnologies, sc-764, 1:200), mouse hybridoma Notch2 (Developmental Studies Hybridoma Bank, University of Iowa, C651.6DbHN, 1:1000) and mouse Cyclin D1(BD Bioscience, #554180, 1:500).

### Tissue/serum analysis of PTER concentrations by gas chromatography and mass spectrometry (GC-MS)

Prostate tissues and serum samples were collected at sacrifice, and kept at −80°C until analysis. Extraction of pterostilbene from prostate tissues was performed following method in Dias *et al*. [10]. Analysis of pterostilbene in the extract was performed by GC-MS according to published procedures [13]. Extraction and analysis of pterostilbene in the serum was performed according to published procedures [13].

### ChIP-Seq experiments and analysis

*Pten*^+/f^ mouse prostates were isolated as described above and 200 mg of tissue was used to perform ChIP-Seq with MTA1 Ab (Bethyl Laboratories, A300-911A, 4 μg) at Active Motif. Reads were aligned to the mouse genome (mm 10). Peak calling was performed using the SICER algorithm at a cutoff of FDR1E-10. The numbers of peaks identified was about 38,000 and 33,000 for MTA1 (Ctrl and PTER, respectively). The ChIP- Seq profiles presented were generated using the UCSC Genome browser.

### Echo MRI analysis

*Pten*^+/f^ male mice on Ctrl-Diet (*n* = 21) and on PTER-Diet (*n* = 30) were individually housed for weekly determination of body weight and body composition starting at 3 months of age until 12 months of age in order to examine any effect of pterostilbene diet supplementation on body weight regulation. Body composition was assessed weekly using magnetic resonance imaging (EchoMRI-900TM, Echo Medical System) to quantify lean mass, fat mass, free water and total water content in conscious mice.

### GEO database analysis

We downloaded the raw expression dataset, GSE41967 [38] from GEO website. The base-2 logarithm transformation was applied to the raw expression data. The transformed expression data were further quantile normalized. Each pair of the selected genes were depicted in the scatter plots. The Spearman rank correlation coefficient was used for correlation assessment to accommodate the skewed distributions. The associated *p* value was calculated based on the null hypothesis of no correlation. We used the Bioconductor and the “normalize.quantiles” function in the “preprocessCore” package to process the expression data.

### Statistical analysis

The histograms were depicted to assess the normality of continuous outcomes. The differences in continuous outcomes between the control and experimental groups were evaluated using the two-sample *t*-test, as well as the Welch's *t*-test when data exhibited unequal variances between the two groups. The Fisher's exact test was used to evaluate the effect of pterostilbene on cancer incidence. In power analysis it was determined that use of 18 mice in each of the vehicle control and pterostilbene injected groups would yield 86% power for confirming a significant association, under the assumption that the vehicle control and pterostilbene injection would be associated with 60% and 10% of adenocarcinoma incidence rates, respectively. Power analysis on mPIN incidence suggested that 7 mice per group and 80 glands per mouse would yield 95% of power for confirming a significant association, under the assumption that glands in the control and pterostilbene groups would be associated with 20% and 12% of mPIN incidence rate, respectively. The experiment of comparing the mRNA levels between the control and experimental groups involved multiple genes. The *p* values were not adjusted for multiple testing because our study of the gene effects was exploratory in nature. All *p* values were two-sided and *p* values less than 0.05 were considered as significant.

## SUPPLEMENTARY MATERIALS FIGURES AND TABLES


