# Supplementary material for: Dietary pterostilbene is a novel MTA1-targeted chemopreventive and therapeutic agent in prostate cancer
Source: Oncotarget. 2016 Mar 1;7(14):18469–84. doi: 10.18632/oncotarget.7841 (PMC4951302; doi:10.18632/oncotarget.7841)
Supplement: Supplementary file 1 [file oncotarget-07-18469-s001.pdf]

Dietary pterostilbene is a novel MTA1-targeted chemopreventive and therapeutic agent in prostate cancer

Supplementary Materials

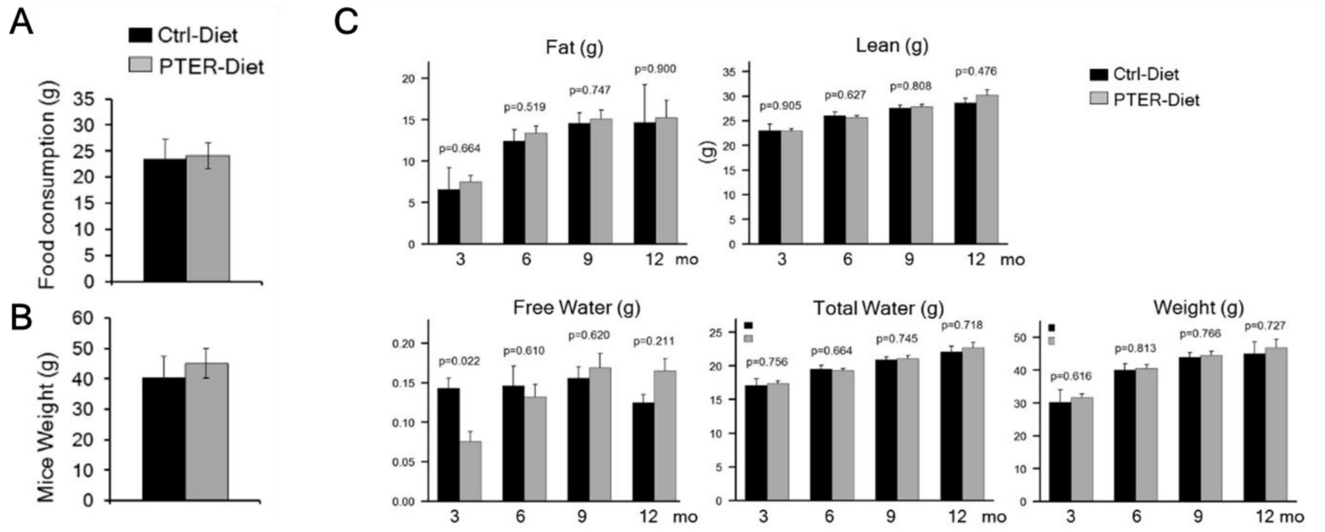

Supplementary Figure S1: Effect of pterostilbene supplemented diet on *Pten*$^{+/f}$ mice. All mice consumed food and water *ad libitum*. There was no significant differences in (**A**) food consumption, (**B**) mice weights between the control (Ctrl-Diet) and PTER-Diet groups. (**C**) EchoMRI measurements for fat, lean, water and weight were comparable for both Ctrl-Diet and PTER-Diet groups at 3−, 6−, 9− and 12− months (mo) of age. Data are mean ± SEM from each age group. 3 mo: Ctrl (n = 2), PTER (n = 6); 6 mo: Ctrl (n =13); PTER (n = 19); 9 mo: Ctrl (n = 18); PTER (n = 26); 12 mo: Ctrl (n = 4); PTER (n = 11). Two accumulations for each mouse. p values were calculated using two-tailed two-sample t-test.

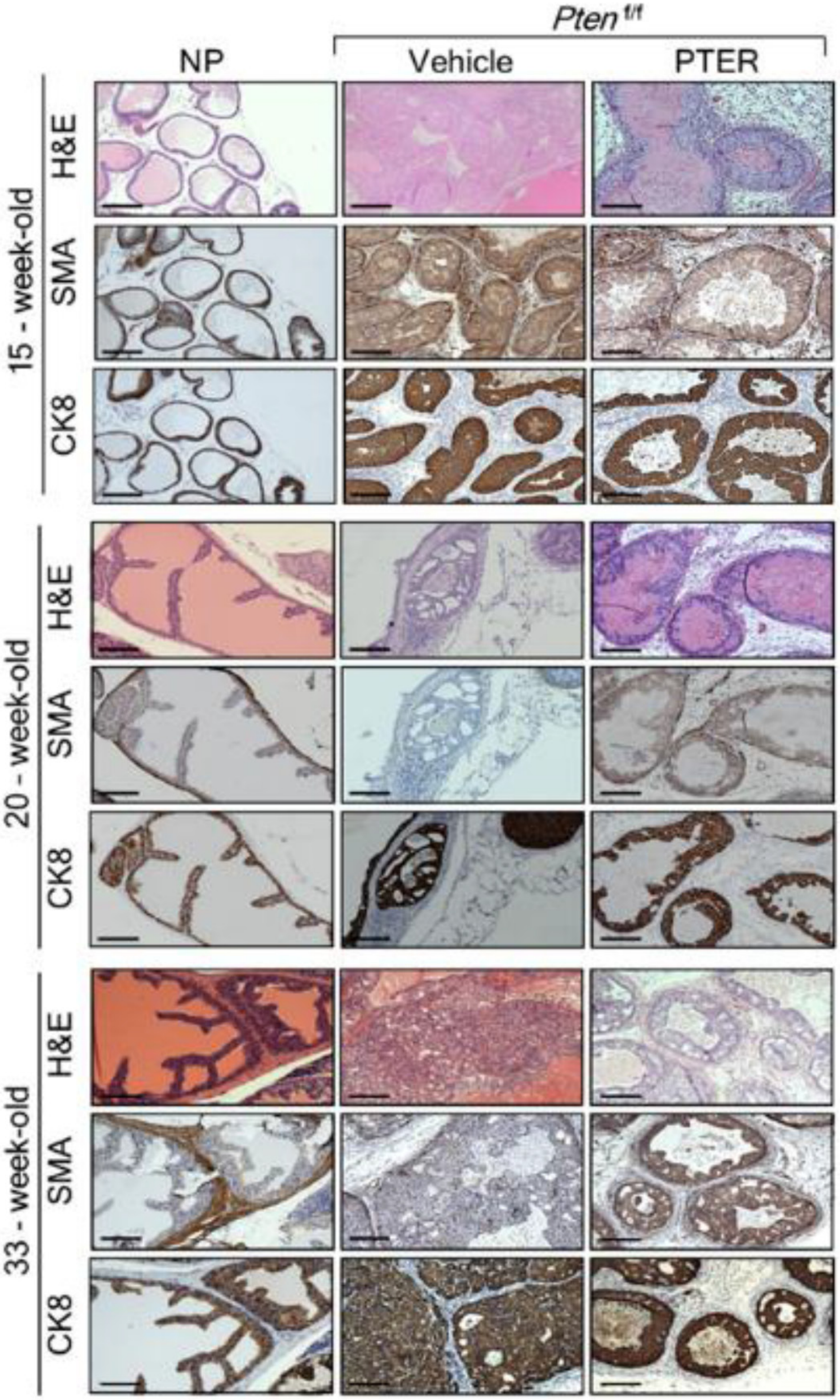

Supplementary Figure S2: Histopathology of *Pten*$^{f/f}$ mice at 15, 20 and 33-weeks age. Comparison of H & E prostate histology (top, each panel) and IHC for SMA (middle, each panel) and CK8 (bottom, each panel) in prostate tissues from representative 15−, 20− and 33− week old Cre-negative mice with normal prostate (NP) and *Pten*$^{f/f}$ mice treated with vehicle and PTER. Scale bars, 100 µm.

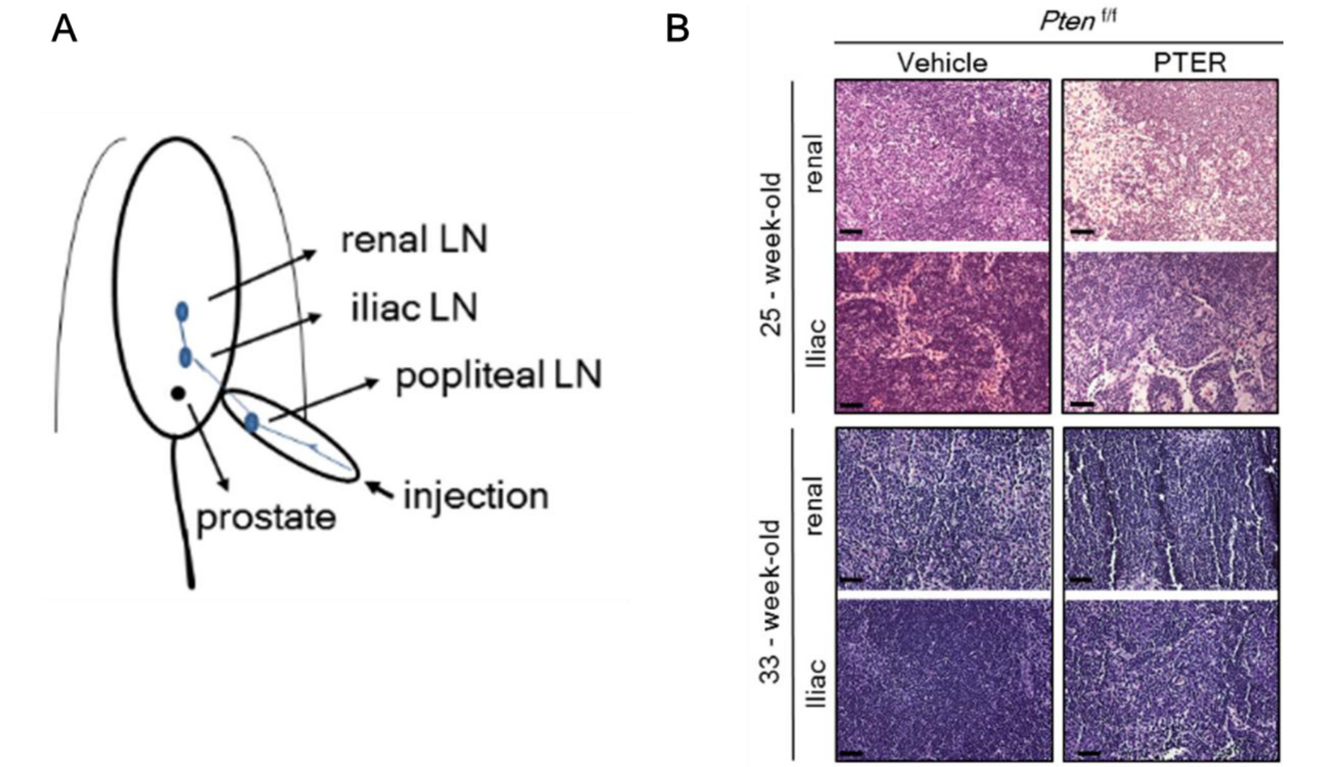

Supplementary Figure S3: Lymph nodes (LN) analyses of *Pten*$^{f/f}$ mice. (**A**) Schematic of the renal and iliac lymph nodes in the mouse. (**B**) Comparison of the H & E LN histology of representative 25-week-old and 33-week-old Pten-null vehicle and PTER-treated mice. Lymph nodes were isolated after injecting the mice subcutaneously at the foot pad (25 µl/ foot pad) with Evans Blue dye and euthanizing after 30 minutes. The Evans Blue dye labels the popliteal LN, which drains centrally to the iliac and renal LNs along the midline. All lymph nodes were benign. Scale bars, 50 µm.

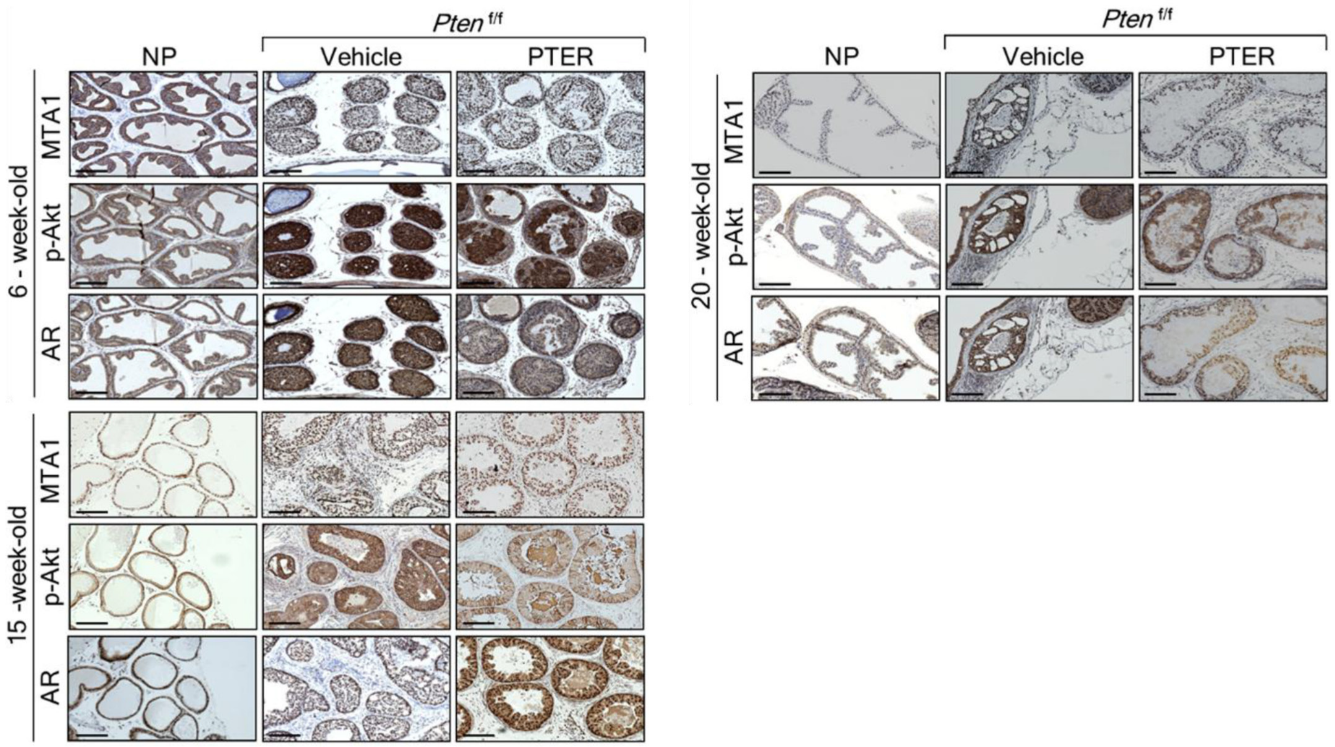

Supplementary Figure S4: IHC analyses of representative 6−, 15−, and 20− week-old *Pten*$^{f/f}$ mice. Comparison of IHC for MTA1 (top, each panel), p-Akt (middle, each panel) and AR (bottom, each panel) from prostate tissues of representative 6−, 15−, and 20− week-old Cre-negative (NP) and *Pten*$^{f/f}$ vehicle and PTER treated mice, respectively. Scale bars, 100 µm.

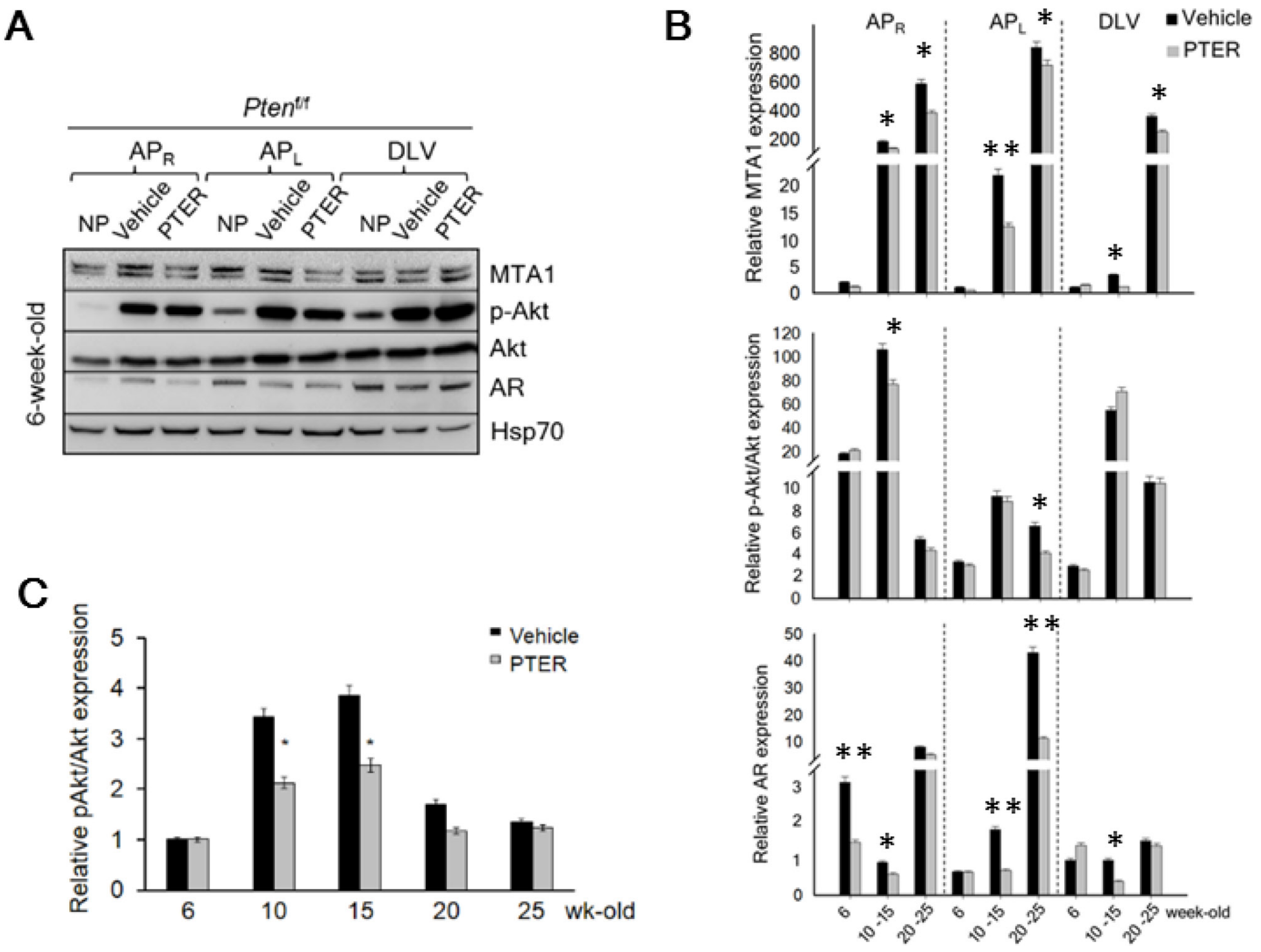

Supplementary Figure S5: Prostate lobe-specific differential impact of pterostilbene (PTER) treatment. (**A**) Representative immunoblots of MTA1, p-Akt, Akt, AR in lysates from prostate lobes isolated from 6-week-old *Cre*-negative (NP) and *Pten*$^{f/f}$ vehicle and PTER treated mice. Hsp70 was used as a loading control. (**B**) Quantitation of MTA1, p-Akt/Akt and AR expression in prostate lobes of *Pten*$^{f/f}$ mice at different ages. (**C**) Quantitation of p-Akt/Akt expression in prostates of *Pten*$^{f/f}$ mice treated with PTER (Figure 6A). Number of mice (n) = 3/group. Values are mean ± SEM. $^{**}p < 0.01$, $^{*}p < 0.05$ (two-tailed, two-sample t-test)

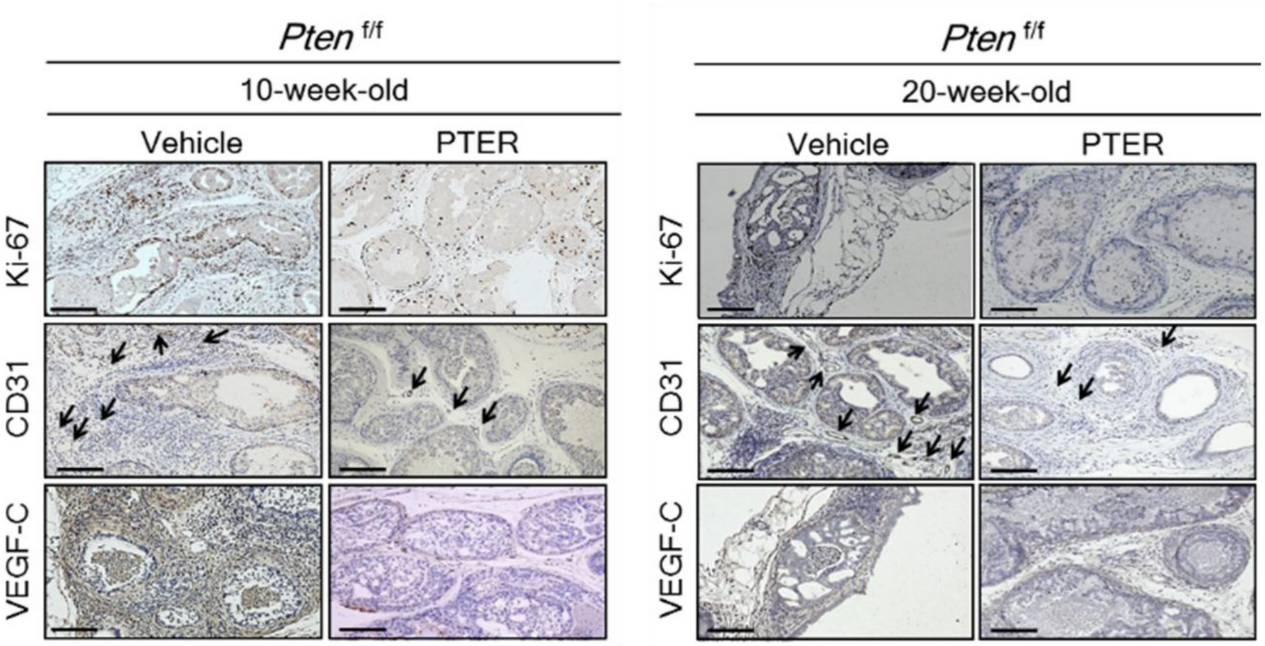

Supplementary Figure S6: Pterostilbene (PTER) reduces proliferation and angiogenesis in prostate tissues of 10−, and 20− week-old *Pten*$^{f/f}$ mice. Representative IHC images for Ki-67 (top, each panel), CD31 (middle, each panel), VEGF-C (bottom, each panel) from *Pten*$^{f/f}$ mice treated with vehicle and PTER are shown. Scale bars, 100 μm (Ki-67, CD31, VEGF-C). Arrows indicate vessels. Also see Figure 8A and 8B.

Supplementary Table S1: List of MTA1 target genes identified in ChIP-Seq analysis from the prostate tissues of *Pten*$^{+/f}$ mice on Control (Ctrl) or pterostilbene (PTER) diet

Gene Name	MTA1 Peak Value	
	Ctrl	PTER
Ets2*	128	75
Ccnd1 (CyclinD1)*	111	79
Notch2*	96	64
Akt1*	90	61
Rela (NF-κB (p65))[#]	83	54
Cdkn1b (p27)*	78	44
Stat3[^]	77	57
Pten[#]	73	45
Smad7[^]	73	47
Gnai2[^]	72	59
Cdkn1a (p21)[#]	66	47
Rnf144a[^]	63	34
Cdh1 (E-cadherin)[#]	61	39
MyD88[^]	60	52
Six3[^]	58	32
Hsp90b1*	55	37
Myc (c-Myc)*	55	34
Hmmr[^]	54	31
Vim[#]	46	28
Vegfc*	43	29
Snai1 (Snail)[^]	38	25
Cry1[^]	37	33
Cdkn2a (p19 ARF)[^]	33	17
Pax5[^]	22	13
Mmp9[^]	20	12
Twist1[^]	19	15
IL-1β[#]	6	4

*Genes newly identified as MTA1 targets by ChIP-Seq analysis in this study.

[#]Genes previously reported as MTA1 targets and identified by ChIP-Seq and validated by qRT-PCR and westerns in this study.

[^]Genes known to be mechanistically regulated by MTA1 and identified by ChIP-Seq analysis in this study.

Supplementary Table S2: Percentage* of normal glands and glands involved in mPIN in *Pten*$^{+/f}$ mice fed with AIN 76A diet (Ctrl-Diet) or diet supplemented with pterostilbene (PTER-Diet) (100 mg/kg diet)

Stage	Glands involved	
	**Ctrl-Diet (*N* = 631)** ***N* (%)**	**PTER-Diet (*N* = 602)** ***N* (%)**
mPIN	126 (20%)	71 (12%)
Normal	505 (80%)	531 (88%)

*Based on histological analysis (H & E) of prostate tissues. *N*, number of total glands from Ctrl-Diet group (n = 7 mice) and PTER-Diet group (n = 10 mice); $p < 0.001$ (Fisher's exact test).

Supplementary Table S3: Incidence* of mPIN, adenocarcinoma and lymph node metastasis in *Pten*$^{f/f}$ mice treated with vehicle or PTER (10 mg/kg bw/day, i.p.)

Stage	Incidence	
	**Vehicle (*n* = 19)** ***n* (%)**	**PTER (*n* = 18)** ***n* (%)**
mPIN	7 (37%)	16 (88%)
Preinvasive Adenocarcinoma	10 (53%)	1 (6%)
Invasive Adenocarcinoma	2 (11%)	1 (6%)
Metastasis (LN)	0 (0%)	0 (0%)

*Stage was defined on the basis of histological analysis (H & E and IHC for SMA and CK8) of prostate tissues and lymph nodes. bw, body weight; i.p., intraperitoneal; mPIN, mouse PIN; LN, lymph node; n, number of mice. $p < 0.01$ (Fisher's exact test).

Supplementary Table S4: Pterostilbene levels in serum and prostate tissues

Mouse model	Route	Dose	Age at sacrifice	PTER (mean ± SEM)	
				**Serum (ng/ml)**	**Tissue(ng/g FW)**
Pten $^{+/f}$	diet	100 mg/kg diet	8–12 mo	8.16 ± 0.11 (n = 26)	7.97 ± 1.15 (n = 17)
Pten $^{f/f}$	i.p	10 mg/kg bw	6-33 wks	11.79 ± 1.75 (n = 25)	30.14 ± 4.72 (n = 7)

Serum and prostate tissues were collected at sacrifice. Pterostilbene (PTER) levels were measured and quantified by GC-MS. Tissues were pooled together from at least three mice at each time point, and PTER was measured when detectable. The values represent mean ± SEM of two repeated measurements of combined tissues within each group. $p < 0.05$ for comparison in serum, $p < 0.01$ for comparison in tissue, two-sided Welch's t -test. FW, fresh weight; bw, body weight; wks, weeks; mo, months; n, number of mice.

Supplementary Table S5: List of primers

Gene	Primers
m **MTA1**	**F**: 5′CACTGGTGCTGAAGCAGGTA 3′<br>**R**: 5′ACTGCTGAGCACACTGGATG 3′
h **MTA1**	**F**: 5′AGCTACGAGCAGCACAACGGGGT 3′<br>**R**: 5′CACGCTTGGTTTCCGAGGAT 3′
m **PTEN**	**F**: 5′GATTACAGACCCGTGGCACT 3′<br>**R**: 5′GGGTCCTGAATTGGAGGAAT 3′
m **Ets2**	**F**: 5′GGGAGTTCAAGCTTGCTGAC 3′<br>**R**: 5′CCCGAAGTCTTGTGGATGAT 3′
h **Ets2**	**F**: 5′GGCTTGGATTCCATTTCTCA 3′<br>**R**: 5′TTGACTCATCACAGCCTTGC 3′
m **Akt1**	**F**: 5′ACTCATTCCAGACCCACGAC 3′<br>**R**: 5′GTCCAGGGCAGACACAATCT 3′
h **Akt1**	**F**: 5′CACACCACCTGACCAAGATG 3′<br>**R**: 5′CACACCACCTGACCAAGATG 3′
m **Notch2**	**F**: 5′CTCCTTCTCCTGCCTGTGTC 3′<br>**R**: 5′AAATGTACTGCCCGTTCAGG 3′
h **Notch2**	**F**: 5′TGTGACATAGCAGCCTCCAG 3′<br>**R**: 5′CAGGGGGCACTGACAGTAAT 3′
m **Hsp90b1**	**F**: 5′CTCACAGAGCCTGTGGATGA 3′<br>**R**: 5′TCTCTGTTGCTTCCCGACTT 3′
h **Hsp90b1**	**F**: 5′TATGTGCGCCGTGTATTCAT 3′<br>**R**: 5′GGGGAGATCATCTGAGTCCA 3′
m **c-Myc**	**F**: 5′GTCAGAGGAGGAACGAGCTG 3′<br>**R**: 5′TCGTCTGCTTGAATGGACAG 3′
h **c-Myc**	**F**: 5′AGCGACTCTGAGGAGGAACA 3′<br>**R**: 5′CTCTGACCTTTTGCCAGGAG 3′
m **CyclinD1**	**F**: 5′GCGTACCCTGACACCAATCT 3′<br>**R**: 5′ATCTCCTTCTGCACGCACTT 3′
h **CyclinD1**	**F**: 5′GATCAAGTGTGACCCGGACT 3′<br>**R**: 5′TCCTCCTCTTCCTCCTCCTC 3′
m **p21**	**F**: 5′TTGCACTCTGGTGTCTGAGC 3′<br>**R**: 5′TCTGCGCTTGGAGTGATAGA 3′
h **p21**	**F**: 5′TGCAGAGAGGTGCATCGTTT 3′<br>**R**: 5′TGG CAGGCAAGGATTTACCCAA 3′
m **p27**	**F**: 5′AACTAACCCGGGACTTGGAG 3′<br>**R**: 5′CCAGGGGCTTATGATTCTGA 3′
h **p27**	**F**: 5′CCGGCTAACTCTGAGGACAC 3′<br>**R**: 5′TTGCAGGTCGCTTCCTTATT 3′
m **β-actin**	**F**: 5′GATCTGGCACCACACCTTCT 3′<br>**R**: 5′GGGGTGTTGAAGGTCTCAAA 3′
h **β-actin**	**F**: 5′CGTGGGCCGCCCTAGGCACCA 3′<br>**R**: 5′TTGGCTTAGGGTTCAGGGGGG 3′

m, mouse; h, human; F, forward primer; R, reverse primer.
